# Increased MMAB level in mitochondria as a novel biomarker of hepatotoxicity induced by Efavirenz

**DOI:** 10.1371/journal.pone.0188366

**Published:** 2017-11-30

**Authors:** Zhimi Tan, Xiaofang Jia, Fang Ma, Yanling Feng, Hongzhou Lu, Jun-O Jin, Dage Wu, Lin Yin, Li Liu, Lijun Zhang

**Affiliations:** Shanghai Public Health Clinical Center, Fudan University, Shanghai, China; University of Missouri Kansas City, UNITED STATES

## Abstract

**Background:**

Efavirenz (EFV), a non-nucleoside reverse transcriptase inhibitor (NNRTI), has been widely used in the therapy of human immunodeficiency virus (HIV) infection. Some of its toxic effects on hepatic cells have been reported to display features of mitochondrial dysfunction through bioenergetic stress and autophagy, etc. However, alteration of protein levels, especially mitochondrial protein levels, in hepatic cells during treatment of EFV has not been fully investigated.

**Methods:**

We built a cell model of EFV-induced liver toxicity through treating Huh-7 cells with different concentrations of EFV for different time followed by the analysis of cell viability using cell counting kit -8 (CCK8) and reactive oxygen species (ROS) using 2',7'-dichlorodihydrofluorescein diacetate (DCFH-DA) and MitoSox dye. Proteomic profiles in the mitochondria of Huh-7 cells stimulated by EFV were analyzed. Four differentially expressed proteins were quantified by real time RT-PCR. We also detected the expression of mitochondrial precursor Cob(I)yrinic acid a,c-diamide adenosyltransferase (MMAB) by immunohistochemistry analysis in clinical samples. The expression levels of MMAB and ROS were detected in EFV-treated Huh-7 cells with and without shRNA used to knock down MMAB, and in primary hepatocytes (PHC). The effects of other anti-HIV drugs (nevirapine (NVP) and tenofovirdisoproxil (TDF)), and hydrogen peroxide (H_2_O_2_) were also tested. Amino acid analysis and fatty aldehyde dehydrogenase (ALDH3A2) expression after MMAB expression knock-down with shRNA was used to investigate the metabolic effect of MMAB in Huh-7 cells.

**Results:**

EFV treatment inhibited cell viability and increased ROS production with time- and concentration-dependence. Proteomic study was performed at 2 hours after EFV treatment. After treated Huh-7 cells with EFV (2.5mg/L or 10 mg/L) for 2 h, fifteen differentially expressed protein spots from purified mitochondrion that included four mitochondria proteins were detected in EFV-treated Huh-7 cells compared to controls. Consistent with protein expression levels, mRNA expression levels of mitochondrial protein MMAB were also increased by EFV treatment. In addition, the liver of EFV-treated HIV infected patients showed substantially higher levels of MMAB expression compared to the livers of untreated or protease inhibitor (PI)-treated HIV-infected patients. Furthermore, ROS were found to be decreased in Huh-7 cells treated with shMMAB compared with empty plasmid treated with EFV at the concentration of 2.5 or 10 mg/L. MMAB was increased in EFV-treated Huh-7 cells and primary hepatocytes. However, no change in MMAB expression was detected after treatment of Huh-7 cells and primary hepatocytes with anti-HIV drugs nevirapine (NVP) and tenofovirdisoproxil (TDF), or hydrogen peroxide (H_2_O_2_), although ROS was increased in these cells. Finally, knockdown of MMAB by shRNA induced increases in the β-Alanine (β-Ala) production levels and decrease in ALDH3A2 expression.

**Conclusions:**

A mitochondrial proteomic study was performed to study the proteins related to EFV-inducted liver toxicity. MMAB might be a target and potential biomarker of hepatotoxicity in EFV-induced liver toxicity.

## Introduction

Chronic administration of the various drugs included under the term highly active antiretroviral therapy (HAART) has changed the prognosis of acquired immune deficiency syndrome (AIDS), and made AIDS a chronic rather than terminal illness [1, 2]. The initial development of these drugs was particularly rapid and focused on clinical efficacy, reduction of mortality before all other considerations [3, 4]. However, as the disease has come under control, there has been a growing emphasis on the long-term adverse effects induced by this therapy.

EFV, a non-nucleoside reverse transcriptase inhibitor, has been used to treat HIV infection since 1998 and evaluated as a successful HAART. Although EFV has considered as a safe drug for the treatment of HIV infection, there are growing concerns that EFV-containing therapies are associated with rash, neuropsychiatric and hepatotoxicity [5]. Up to 8–10% of HIV patients treated with EFV exhibit increases in liver enzymes, among them about 1.3% patients developed severe hepatotoxicity (grade 3 to 4 elevations in aspartate aminotransferase and/or alanine aminotransferase); toxicity that may result in the treatment being discontinued [6–9]. Moreover, previous studies found that inter-individual variability in drug metabolizing enzymes due to genetic polymorphisms which lead to supratherapeutic drug concentration [10, 11]. In addition, clinically useful concentrations of EFV induce cell apoptosis in human hepatoblastoma Hep3B cells [12]. Although these EFV-induced events have been characterized, the cellular and molecular mechanisms underlying these detrimental effects of EFV remain largely unknown.

The mitochondrion is a major target of EFV-induced cytotoxicity and a wide variety of mechanisms are involved. Treatment of EFV (25 or 50μM) induces decreases in mitochondrial membrane function, intracellular ATP levels and complex I–dependent respiration. It also promotes increases in the mitochondrial superoxide and ROS production, causing oxidative stress in the mitochondria [13–15]. Moreover, treatment of EFV decreases O_2_ consumption resulting in mitochondrial dysfunctions. In addition, EFV-induced reduction in energy production triggers a compensatory mechanism mediated by the enzyme adenosine monophosphate–activated protein kinase (AMPK) [12]. Furthermore, EFV induces autophagy and, in particular, mitophagy [16] and endoplasmic reticulum (ER) stress through a process that involves mitochondrial interference [17]. According to these reports, it is sure that EFV inhibits mitochondrial function. However, the alterations of protein expression and protein-protein interactions in mitochondrion during EFV treatment have not been investigated.

Proteomic analysis, providing global protein information, is of great value for toxicological studies [18, 19]. In toxicological studies, the clues to the mechanisms eliciting toxicity can be determined by the approach of analyzing and comparing the differentially expressed proteins. The differentially expressed proteins can subsequently be used as potential biomarkers for monitoring toxicity.

The present study aimed to investigate the hepatotoxicity mechanism of EFV in Huh-7 cells through a proteomic strategy. To determine mitochondrial function, we measured ROS production in EFV-treated Huh-7 cells (a human hepatoma cell line) at different concentrations and time points. After defining the effective concentration of EFV for mitochondrial stimulation, we separated stimulated mitochondria and analyzed the alteration of proteins then assessed the role of mitochondria proteins in the toxic response to EFV. This study provides the basis for understanding the underlying mechanisms of hepatotoxicity of EFV and indicates a potential biomarker to monitor the liver toxicity during EFV treatment.

## Materials and methods

### Cells, reagents, and drugs

Two kinds of cells including Huh-7 cells (a human hepatoma cell line) and primary hepatocytes were used. Huh-7 cells were from the Cell Bank of the Chinese Academy of Sciences (Shanghai, China). Two bottles of primary human hepatocytes (PHC) (one from male and one from female) were bought from Research Institute for Liver Diseases (Shanghai) Co. Ltd Company (Shanghai, China). Both PHC have normal CYP2B6 and CYP3A4 (the main metabolic enzymes for EFV). Huh-7 cells were cultured in Dulbecco's modified eagle medium (DMEM) (High glucose) containing 10% FBS and penicillin, streptomycin, and glutamine. Two bottles of primary hepatocytes were mixed in culture medium like Huh-7 according to manufacturer's advice [20], and used for experiments within 24 h.

All reagents used for culture were obtained from Gibco (Invitrogen, Carlsbad, California). EFV, Nevirapine (NVP, an anti-HIV drug with hepatotoxicity), and Tenofovir disoproxil fumarate (TDF, another anti-HIV drug, with no hepatotoxicity) were provided by Merck (Whitehouse Station, New Jersey, USA). MitoSOX Red mitochondrial superoxide indicator, for live-cell imaging was from Thermofisher Scientific (Waltham, MA, USA). Rotenone (a complex I inhibitor) and H_2_O_2_ (a chemical reagent which increases oxidant stress) was provided by Sinopharm Chemical Reagent Co., Ltd (Shang, China). Reagents for proteomics were from General Electric (GE) (Fort Myers, Florida, USA). Hochest 33342, Reactive Oxygen Species Assay Kit (DCFH-DA), and Cell Counting Kit-8 (CCK-8) was from Beyotime Company (Jiangsu, China). EFV, NVP and TDF were dissolved in methanol, methanol and water to make solutions with concentration of 2.5 and 10 mg/mL for EFV, 7.0mg/mL for NVP, and 5.0mg/mL for TDF according to previously descripted [16, 21, 22].

### Human subjects

Eleven patients with HIV who required liver biopsy diagnosis provided written consent for research use of the remaining biopsy material. These patients included five from EFV-treated, four from untreated HIV patients and two from protease inhibitor- (PI) treated patients (Table 1). All the patients tested positive for HIV and negative for hepatitis C virus (HCV) and hepatitis B virus (HBV). Their CD4 and CD8 blood cell counts had no significant differences between the two groups. The study protocol was approved by the Ethics Committee of Shanghai Public Health Clinical Center.

**Table 1:** Clinical information of patients involved in this study.

NO.	Drugs	Age (y)	ALT (U/L)	AST (U/L)	ALP (U/L)	GGT (U/L)	HBV	HCV	Syphilis	TB	CD4 (/mm^3^)	CD8(/mm^3^)	Liver diseases	HIV
With HAART (including EFV)														
1	TDF+3TC+EFV	40	26	51	89	42	N	N	N	N	22	190	Liver Cirrhosis	Y
2	D4T+3TC+EFV	29	28	51	137	145	N	N	N	N	442	1011	Liver abscess (tuberculosis)	Y
3	TDF+3TC+EFV	51	28	36	129	72	N	N	N	N	96	1011	Liver cancer	Y
4	D4T+3TC+EFV	32	38	80	116	188	N	N	N	N	303	336	Hepatitis C cirrhosis	Y
5	3TC+TDF+EFV	33	50	80	60	45	N	N	N	N	360	600	Chronic hepatitis with severe steatosis CH-G1S2	Y
Without HAART or EFV														
6	Not	48	54	36	75	73	N	N	N	N	115	220	Multiple hepatic cysts	Y
7	Not	33	22	15	58	19	N	N	N	N	400	1118	Hepatic lesions	Y
8	Not	63	50	48	51	26	N	N	N	N	166	918	Cirrhosis	Y
9	Not	41	100	202	65	23	N	N	N	N	381	545	Cirrhosis	Y
10	TDF+3TC+LPV	42	22	42	98	38	N	N	N	N	141	261	Cirrhosis	Y
11	TDF+3TC+LPV	36	51	111	97	70	N	N	N	N	68	214	Cirrhosis	Y

ALT: alanine aminotransferase, AST: Aspartate transaminase, ALP: alkaline phosphatase, GGT: glutamyl transferase, HBV: Hepatitis B virus, HCV: hepatitis C virus, HIV: human immune deficiency virus, CD4: cluster of differentiation 4, CD8: cluster of differentiation 8, Y: Yes, N: Not.

### Cell model construction and evaluation

#### Cell viability

To make a EFV-induced hepatotoxicity cell model, we treated Huh-7 cells with EFV at the concentrations of 2.5 or 10 mg/L for different time (0, 0.5, 1.0, 1.5, 2.0, 2.5, 3.0, 6.0, and 12.0 h). Cell viability was detected by CCK-8 according to Beyotime Company’s instruction to select suitable EFV concentration and treating time.

#### Reactive oxygen species (ROS) production

Since mitochondrion is one of the main targets of EFV-induced hepatotoxicity. Increased production of ROS was generally detected during mitochondrial dysfunction [13–15]. So in this work, we used ROS as an indicator of mitochondrial dysfunction and EFV-induced hapatotoxicity [22]. Briefly, Huh-7 cells were seeded in a black 24-well plate, and treated with EFV (0, 2.5, 5.0 or 10 mg/L) for 0.25, 0.5, 0.5, 1.0, 1.5 and 2.0 h to analyze concentration and time-dependence of EFV toxicity. To make sure EFV-induced ROS production within 2 h, Huh-7 cells or human primary haptocytes were treated with EFV (0, 2.5, or 10 mg/L), two other anti-HIV drugs (NVP (7 mg/L), or TDF (5 mg/L)), or two positive controls (H_2_O_2_ (500 μM), or rotenone (10 μM) (Complex I inhibitor)) for 2 h. The treated cells were incubated with DCFH-DA (2',7'-dichlorodihydrofluorescein diacetate, 2.5μM) for total ROS production detection. After incubated for 20minutes with DCFH-DA, the cells were washed for three times with PBS, and detected by flow cytometry technology with excitation wavelength of 488 nm and emission wavelength of 525 nm.

To detect mitochondrial ROS, specific mitochondrial probe MitoSOX was used. Briefly, Huh-7 cells or Human primary hapatocytes were treated with EFV (2.5 or 10 mg/L), NVP (7 mg/L), TDF (5 mg/L), H_2_O_2_ (500 μM), or rotenone, and incubated with probe MitoSOX for 10 minutes for 2 h, and detected by flow cytometry technology or confocal microscopy.

### Mitochondrion enrichment and verification

Differential centrifugation in conjunction with aqueous two-phase partition was used for mitochondrion isolation as previously described [23]. Briefly, all steps were carried out at 4°C. After EFV treatment for 2 h, the cells (5.0 ×10^8^) were washed three times with PBS, scraped using a plastic cell lifter, and broken in 1.0 mL solution containing 0.2 mM EDTA and 1.0 mM NaHCO_3_ using a glass homogenizer. The nuclei and unbroken cells were removed by centrifugation at 200 g for 10 min; the supernatant was collected, and centrifuged for 30 min at 25,000 rpm. The cell pellets were resuspended in 1 mM NaHCO_3_ in an approximate ratio of 1 ml per 5.0 ×10^8^ cells and used for mitochondrion separation by two-phase systems [24]. Suspended cell pellet (2 g) was added to the top of 14 g of a dextran-polyethylene-glycol mixture (6.6% Dextran T500, 6.6% PEG 3350, 0.2 M K_3_PO_4_, pH 7.2). After mixing by shaking 40 times, the tube was centrifuged for 5 min at 750 g. The mitochondrion-enriched down phase and plasma membrane-enriched up phase were collected and purified again as before. These two phases were diluted 5-fold with 1.0 mM sodium bicarbonate, and centrifuged at 100,000 g for 30 min in a SW32 rotor. The pellets were collected and used for purity check and proteomics analysis.

To characterize the contents of these two fractions, 25μg of protein extraction was analysed by WB with antibodies against known molecular markers of mitochondria and plasma membrane (PM) (Na^+^/K^+^-ATPase for the PM and prohibitin for the mitochondrial apparatus, respectively).

### Two dimension electrophoresis (2DE) and image analysis

The first dimension of 2DE was performed on an IPGphor iso-electronic focusing system (General Electric (GE) Company, USA). Extracted mitochondrion proteins (250 μg) were mixed with IEF sample buffer containing 8 M urea, 2 M thiourea, 65 mM DTT, 20 mM Tris-base, 4% CHAPS, 0.5% IPG buffer, pH 3–10 NL and a trace of bromophenol blue to a total volume of 350 μL, and applied to IPG DryStrips (pH 3–10 NL; 180×3×0.5 mm). IEF was performed at 20°C under the following conditions: 50 V for 14 h, 500 V for 1 h, 1000 V for 1 h, 8000 V gradient for 2250 Vhr, and 8000 V for 6 h up to 51.7 KVh. Following IEF, the gel strips were equilibrated twice, each for 15 min in equilibration buffer (pH 8.8) containing 6 M urea, 50 mM Tris-HCl, 30% glycerol and 2% SDS in which 0.2% DTT was added into the first step equilibration buffer and 3% iodoacetamide was added into the second. The second dimensional run was carried out on a SDS-PAGE vertical slab 14% separation gel against a series of molecular weight markers on Bio-Rad Protein II electrophoresis apparatus. Separate gels were run at constant current of 30 mA/gel. After completion of the 2DE, gels were stained using Coomassie brilliant blue G-250. The 2DE gels were scanned via Imagescaner (GE Company, USA) in transmission mode.

Three images from each test group were analyzed against three control images using ImageMaster software (GE Company, USA). Differences of > 1.5 or < 0.66 -fold in the volume of a test spot compared to the volume of the same spot in the control gel were considered to be significant. For normalization purposes, 48 marker protein spots were selected.

### Protein identification

The differential protein spots were cut out and in-gel digested as published [25, 26]. The digested peptides were analyzed via nanoLC-ESI mass spectrometry (Esquire HCT, Bruker, Germany). The tryptic peptide mixtures were injected onto a C18 μm–pre-column (300 μm id × 5 mm, 5 μm, PepMap™) (Dionex, USA) at a flow rate of 20 μL/min using an Ultimate 3000 (Dionex, USA). After being desalted by the pre-column, the peptides were then added to a C-18 reverse-phase nanocolumn (75 μm id × 15 cm length, 3 μm, PepMap™) (Dionex, USA). The flow rate was 300 nL/min with a continuous gradient consisting of 2–40% acetonitrile (ACN) and 0.1% formic acid (FA). The eluted peptides from the reversed-phase nanocolumn were injected online with a PicoTip emitter nanospray needle (New Objective, Woburn, MA, USA) for real-time ionization and peptide fragmentation in a HCT mass spectrometer. The MS/MS data was analyzed using the MASCOT 2.0 program (MatrixScience, Boston, MA, USA) to search SWISS-PROT database. Search parameters were set as follows: (a) enzyme, trypsin: allowance up to one missed cleavage; (b) mass tolerance: 1.2 Da, and MS/MS mass tolerance: 0.6 Da; (c) fixed modification: carbamoylmethylation (C); (d) variable modification: oxidation (M); (e) species: *Homo sapiens*; (f) auto hits allowed (only significant hits were reported). Proteins were identified on the basis of peptides with ion scores exceeding the threshold (*p* < 0.05), which indicated identification at the 95% confidence level for these matched peptides. Proteins identified by more than four peptides were accepted without manual check. Proteins identified with less than three peptides were manually inspected to make sure at least one peptide had four or more continued y-or b-series ions (e.g., y2, y3, y4, y5).

### Quantitative reverse transcription polymerase chain reaction (qRT-PCR)

Primers were designed using Primer 3 software (Applied Biosystems, Foster City, CA, USA). Total RNA was extracted from EFV-treated Huh-7 cells using TRIzol reagent, as described previously [27]. For ALDH3A2 detection, MMAB shRNA transfected Huh-7 cells were cultured for 48 h, and total RNA was extracted. Subsequently, cDNA was synthesized via RT-PCR with the SuperscriptⅡkit (Life Technologies, Karlsruhe, Germany). Real-time Q-RT-PCR was performed on a 25 μl reaction mixture containing 700 nM forward and reverse primers, 80 nmol template and 1 × Sybr Green reaction mix (Applied Biosystems, Foster City, CA, USA). Sybr Green fluorescence was determined with the ABI PRISM 7500 detection system (Applied Biosystems, Foster City, CA, USA). The mRNA expressions of genes were normalized against that of the control gene (Glyceraldehyde 3-phosphate dehydrogenase (GAPDH)). The primers of four differentially expressed genes used in study are shown in Table 2.

**Table 2:** Comparison of mRNA and protein levels of the four genes with mitochondrial location.

spot	Protein name (Gene name)		Primer	mRNA		Protein	
				2.5/C	10/C	2.5/C	10/C
Spot 2	Glycerol-3-phosphate dehydrogenase (GPD2)	sense	CTCCCCATTTATCAGCTCCA	3.2	3.6	↓3.7	<10
		antisense	CCGTGCATCGTTATGTTGTC				
Spot 6	Cob(I)yrinic acid a,c-diamide adenosyltransferase (MMAB)	sense	AAACGGGAGACAAAGGGTTT	3.2	16.2	>10	>10
		antisense	TGCAATGTGCACTGGATTTT				
Spot 10	NADH dehydrogenase [ubiquinone] iron-sulfur protein 8, mitochondrial precursor (NDUFS8)	sense	ACCCGCTATGACATCGACAT	1.01	1.37	>10	>10
		antisense	CACTTGTCCCCGTTGTTGAG				
Spot 13	Nesprin-2 (SYNE2)	sense	AGGGTGGCCATACGTAAACA	0.98	1.87	>10	>10
		antisense	TGTTGCTGGCCTTGATCAAC				

### Western blotting (WB)

WB was performed using mitochondrial extracts or whole cell extracts from Huh-7 cells with or without MMAB knockdown, or from primary hepatocytes treated with EFV, NVP, TDF or H_2_O_2_. Proteins were separated by electrophoresis in SDS-12.5% polyacrylamide gel and transferred to PVDF membrane (Millipore company, Boston, Massachusetts, USA) as described elsewhere [27]. After blocking in 10% defatted milk for 2 h at room temperature or overnight, blots were incubated for 2 h at 37°C or overnight with specific primary antibodies (mouse anti-Na^+^/K^+^-ATPase (1:5,000; Abcam, Cambridge,UK), rabbit anti-prohibitin (dilution: 1:100; Abcam), mouse anti-SERA (1:500, Santa Cruz Biotechnology, California, USA), rabbit anti-MMAB (1:1000; Abcam, Cambridge, UK), mouse anti-β-Actin (1:1000; Santa Cruz Biotechnology, California, USA)), and mouse anti-ALDH3A2 (1:100, Santa Cruz Biotechnology, California, USA). After three washes with TBST (TBS+Tween), blots were incubated for 1 h at room temperature with secondary antibodies (goat anti-mouse IgG-HRP (1:2000, Santa Cruz Biotechnology, California, USA) and goat anti-rabbit IgG-HRP (1:2000, Santa Cruz Biotechnology, California, USA)). After further washes, the immune complexes were revealed by enhanced chemiluminescence and detected by X-ray films. Each experiment was performed in triplicate. Finally, the densitometric analysis of the films was performed through Image J software (http://rsb.info.nih.gov/ij).

### Immunohistochemistry and semi-quantitation of MMAB expression

Immunohistochemistry was performed as described previously [27]. Sections (5 µm) of paraffin-embedded liver tissues from the eleven HIV-infected patients were de-paraffinized, hydrated, and washed three times in PBS. Subsequently, the slides were incubated overnight at 4°C with rabbit anti-MAAB (dilution 1:1000) in a humidified chamber. The slides were washed in PBS three times, incubated with horseradish peroxidase-conjugated anti-rabbit antibody, and detected using a liquid 3, 3'-diaminobenzidine (DAB) staining kit (Gene Tech, Shanghai, China), counterstained with hematoxylin–exon, dehydrated, mounted in Permount (Fisher Scientific), and imaged digitally by light microscopy using an Olympus BX40 equipped with a logenE PAS9000.

To semi-quantitatively analyze the expression of MMAB in liver tissue, each slide was randomly imaged 10 times in 400-fold magnification. The MMAB-positive cells were counted in each image. The ratio of the average number of positive cells from 5 patients treated with EFV (total of 50 images) to that of five patients without HAART (total of 50 images) was considered to be the proportional change of MMAB expression.

### Bioinformatics

The theoretical isoelectric point (pI) and molecular weight (MW) were extracted through the Mascot software. The subcellular location and function of the identified proteins were elucidated by Uniprotkb/Swiss-Prot including integrated Gene Ontology Database (http://www.uniprot.org/uniprot). For the pathway analysis, gene names were used to search KEGG database (http://www.kegg.jp). For amino acid pathway analysis, we focused on beta-Alanine metabolism.

### MMAB small hairpin RNA (shRNA) transfection and function analysis

MMAB shRNA transfection was performed according to the previous report [28]. Briefly, the shRNA specific for MMAB (shMMAB) was designed according to Genebank accession number NM_052845 (Uniprotkb number of MMAB_HUMAN) wherein the hairpin RNA sequence is GGAGGCTCACTTAAAGTAT. The Interference vector GV248 was purchased from Shanghai Genechem Company (Shanghai, China). Huh-7 cells were transfected with MMAB-shRNA or its non-targeting control shRNA at the concentration of 0.8 or 2 μg per well (24 or 6 well-plate array) for 6 h in serum-free culture medium. Fetal bovine serum was then added to a final concentration of 10%. 48 h later, the cells were treated with EFV or H_2_O_2_ or Rotenone, and used for ROS analysis, treated with EFV, or lysed and used for WB detection and real-time RT-PCR to verify the effect of shMMAB, expression of ALDH3A2 or for amino acid analysis. For amino acid analysis, the cells were disrupted through freezing and thawing three times, followed by grinding in liquid nitrogen. Then the broken cells were centrifuged at 12000 rpm at 4°C for 15 min, and the supernatant was collected. An equal volume of 10% Trichloroacetic acid was added to the above supernatant. After 60 min at 4°C, the sample was centrifuged at 15000 rpm at 4°C for 30 min. The supernatant was withdrawn and adjusted to pH 1.7 to 2.2 using 8 mol/L NaOH (usually 24 μL 8 mol/L NaOH for 800 μL samples), then centrifuged at 15000 rpm at 4°C for 15 min and filtered using 0.45 μg filter membrane. The filtrate (with volume more than 200 μL) was freeze-dried and re-dissolved with 400μL hydrochloric acid (0.02 mol/L). After centrifugation at 15000 rpm at 4°C for 15 min, the supernatant was withdrawn. 50 μL was injected into an amino acid analyzer (Hitachi L8500, Tokyo, Japan). For real-time RT-PCR analysis of ALDH3A2, the sense and antisense primers are ACAATACCCAGGAAAGTG, and GAAGGTGCTAACAAACTCA, respectively.

### Data statistic analysis

For each experiment, three replicates were performed. Statistical analysis was carried out by unpaired t tests (GraphPad Primer 5 Software, La Jolla, CA). Statistical difference was shown in the follow: ns, no difference; *, P < 0.05; **, P < 0.01; and ***, P < 0.001.

## Results

### EFV treatment reduced cell viability

**Fig 1:**
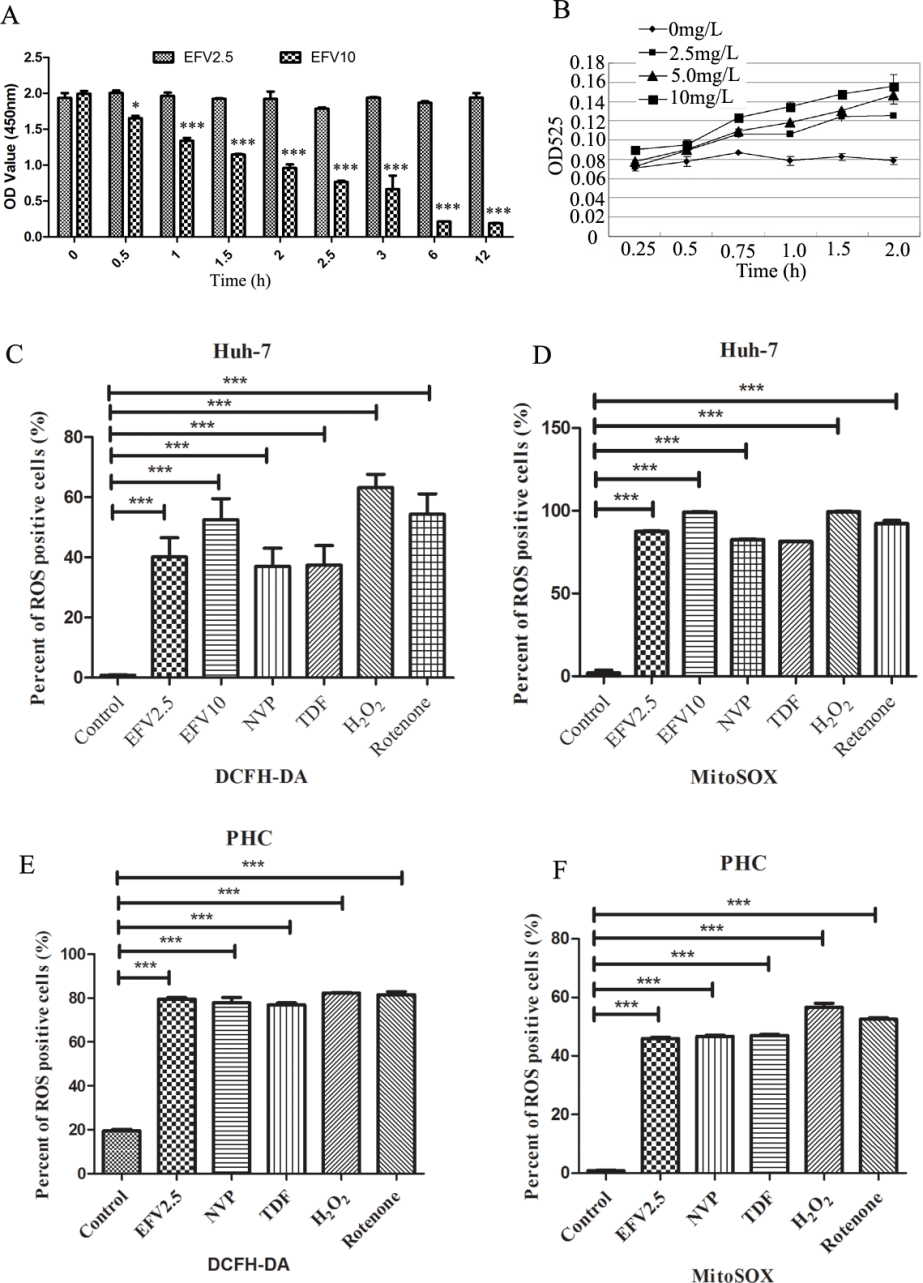
EFV inhibited cell proliferation and induced ROS production. A) Huh-7 cells were incubated with EFV (2.5 and 10 mg/L) for 0, 0.5, 1.0, 1.5, 2.0, 2.5, 3.0, 6.0, and 12.0 h, and detected by CCK8 kit. B) Huh-7 cells were incubated with the indicated concentrations of EFV for the indicated times. Production levels of ROS were detected with DCFH-DA probe. The cells were treated with EFV, NVP, TDF, H_2_O_2_ or rotenone for 2 h and analyzed the expression levels of ROS by flow cytometry. C) Total ROS was detected by DCFH-DA in Huh-7 cells. D) Mitochondrial ROS was detected by mitoSOX in Huh-7 cells. E) Total ROS in primary hepatocytes. F) Mitochondrial ROS levels in primary hepatocytes are shown. Data are the average of three independent experiments (total n = 3). Data shown are the mean ± SEM. ns, no significant difference, * *p < 0*.*05* ** *p < 0*.*01*, and ***p<0.001.

To build an EFV-induced hepatotoxicity cell model, we detected the effect of EFV on cell viability of Huh-7 cells with different concentration of EFV for different time by CC8 kit. As shown in Fig 1A, during the 12 h experiment period, no significant difference was observed in viability of Huh-7 cells treated with 2.5mg/L EFV. However, 10mg/L EFV inhibited viability of the cells, which showed 48% and 10% of viabilities after 2 and 6 h treatment, respectively. So in this work, subsequent experiments were performed within 2 h.

### EFV induced ROS production in Huh-7 cells and primary hepatocytes

Previous studies have shown that EFV inhibits mitochondrial function, resulting in increased production of ROS [16, 22]. To determine effective concentration and time for mitochondria stimulation, we measured the level of ROS production in Huh-7 cells after treatment of EFV. Huh-7 cells were cultured with 1.25, 2.5, 5 and 10 mg/L of EFV for 2 h and then ROS levels were measured in the culture medium. Concentrations of 2.5 mg/L to 10 mg/L EFV significantly promoted increases in the ROS production (Fig 1B). Next, we examined time-dependency of the effect of EFV in ROS production. EFV at 2.5 mg/L induced substantial elevation in a time-dependent manner and 5 and 10 mg/L of EFV showed a similar pattern of stimulation of ROS (Fig 1B). These data indicate that treatment with EFV can promote ROS production in a dose and time-dependent manner in Huh-7 cells. To make sure EFV-induced ROS production increase within 2 h, we used positive control H_2_O_2_ and rotenone, and other anti-HIV drugs (NVP and TDF) to treat Huh-7 cells and primary hepatocytes, and found after 2 h treatment, EFV induced total (Fig 1C) or mitochondrial ROS (Fig 1D, S1 Fig) production similar like H_2_O_2_ and rotenone, and stronger than NVP and TDF in Huh-7 cells. Similarly, EFV induced upregulation of total (Fig 1E) or mitochondrial ROS (Fig 1F) production in primary hepatocytes, with similar increases of EFV 2.5, NVP, TDF, H_2_O_2_ or rotenone treatment.

### EFV treatment in Huh-7 cells modulated expression of 4 different proteins in mitochondria

**Fig 2:**
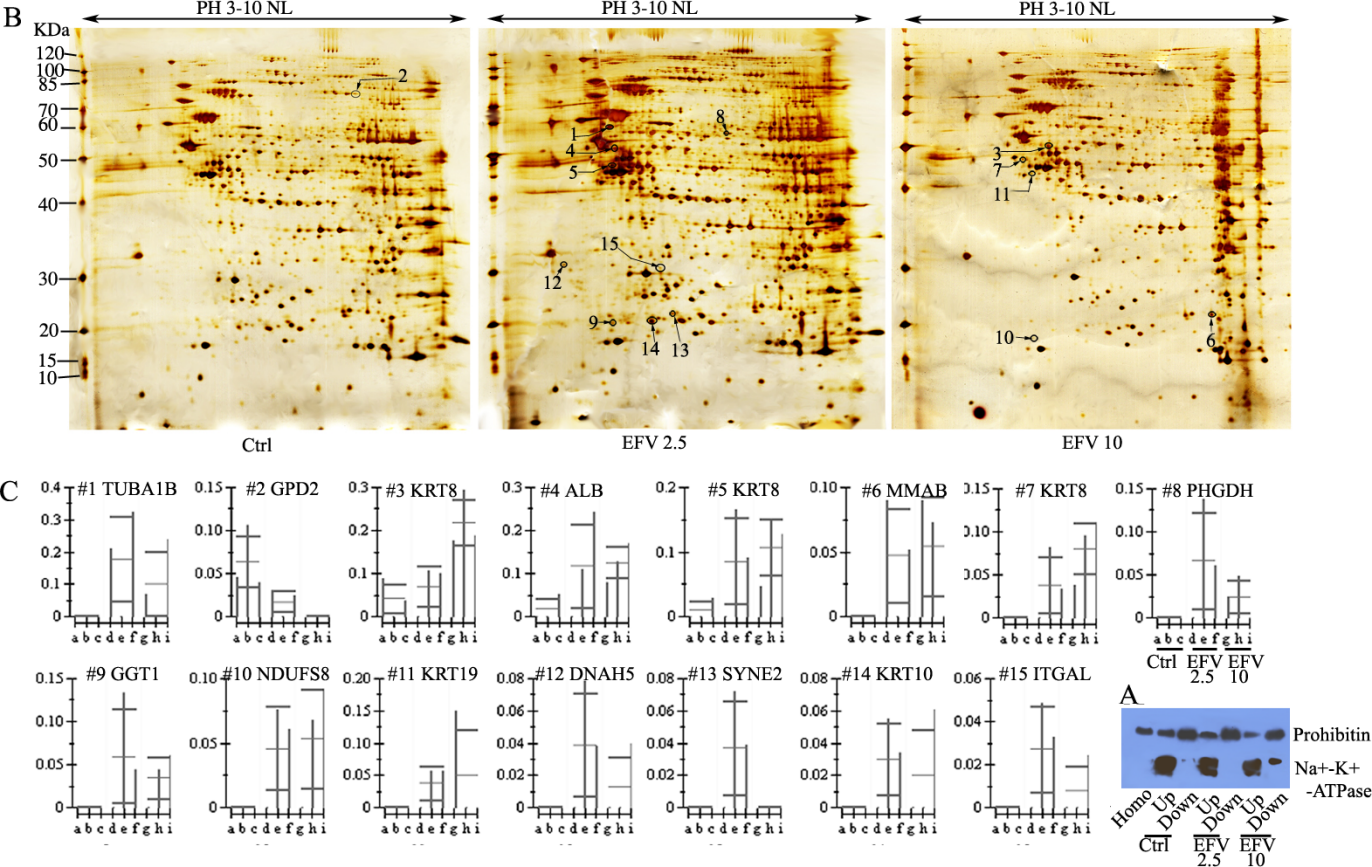
EFV induces alteration of the expression of 15 proteins in Huh-7 cells. Huh-7 cells were treated with 2.5 and 10 mg/L EFV for 2 h. A) Enrichment of mitochondrial proteins is shown. The blots were probed with antibodies against organelle-specific proteins: anti-prohibitin for mitochondria, anti-Na+/K+-ATPase for plasma membrane; Homo, up and down mean homogenization, upper phase, and lower phase differences from control, EFV2.5 and EFV 10. B) Prohibitin-enriched upper phase was collected as mitochondria and analyzed by 2DE separation. The up-regulated proteins are shown. There were none down-regulated. C) Differentially expressed proteins were analyzed by Imagemaster software and shown as a bar graph. Data are representative of, or the average of, analyses of three independent experiments. The columns a, b & c represent control, d, e & f represent EFV 2.5, and g, h & i represent EFV 10.

Since EFV induced the production of ROS, we next examined the change of mitochondrial proteins by EFV treatment. Huh-7 cells were cultured with 2.5 and 10 mg/L EFV for 2 h and harvested. Using a two-phase partition method, we enriched mitochondrial proteins and determined the enrichment of mitochondria proteins by WB, including up and down phase. As shown in Fig 2A, the down phase contained higher amounts of prohibitin, a specific marker of mitochondria, compared to the up phase. Moreover, the down phase showed low levels of Na^+^/K^+^-ATPase, a critical biomarker of plasma membrane, whereas strong signal of Na^+^/K^+^-ATPase were detected in the up phase. These data indicated that we efficiently enriched mitochondria proteins by using two-phase partition.

In order to explore the signature molecular biomarkers of EFV toxicity in Huh-7 cells, a 2DE-MS-based proteomic method was performed to analyze three groups’ samples. EFV 0, EFV 2.5 and EFV 10. 250 μg enriched mitochondria proteins were loaded on 18-cm-2-D gels (pI 3–10 NL) and stained with silver nitrate. As shown in Fig 2B, treatment of EFV 0, 2.5 and 10 gave 531±20, 543±30, 515±15 protein spot sizes respectively, as measured by ImageMaster software analysis. Moreover, according to statistical analysis with significant *p* values (*t*-test, *p<0*.*05*) and an intensity change (ratio of > 1.5 or <0.66 –fold), a total of 15 differentially expressed protein spots were detected at EFV 2.5 and EFV 10 compared with the control group (Fig 2B). Furthermore, the quantitative analysis of images showed that the expression levels of 14 proteins were significantly increased by EFV, whereas 1 protein was decreased in expression level compared to control group (Fig 2C). In addition, we characterized the 15 protein spots by LC-MS/MS analysis, and found that 13 of them were non-abundant (Table 3). Moreover, 4 of the protein spots with altered expression levels were identified as mitochondrial proteins (Table 3). Among these proteins, only spot 9 was identified by one peptide with mascot score of 75 (S2 Fig and S1 Table) and one was identified by two peptides (S1 Table).

**Table 3:** List of protein spots identified by 2DE as differentially expressed after EFV treatment.

Spot	Name	Accession Number (Gene Name)	MW (Da)	PI	Score	Matched Peptides (No.)	Regulation in EFV Treatment (2.5、10)	emPAI	Location	Function	Pathway*
1	Tubulin alpha-ubiquitous chain	TBAK_HUMAN (TUBA1B)	50804	4.94	770	15	>10、>10	1.26	cytoplasm; cytoskeleton	binding; structural	10; 12; 13
2	Glycerol-3-phosphate dehydrogenase, mitochondrial precursor	GPDM_HUMAN (GPD2)	81296	7.23	603	22	0.3、<-10	0.37	mitochondrion	Binding; enzyme;	15
3	Keratin, type II cytoskeletal 8	K2C8_HUMAN (KRT8)	53671	5.52	941	18	1.7、5.3	1.59	cytoplasm/keratin filament/nuclear matrix/nucleoplasm	binding; structural	ND
4	Serum albumin precursor—Homo sapiens	ALBU_HUMAN (ALB)	71317	5.92	638	17	6.8、7.3	0.8	Secreted	antioxidant activity; binding;	ND
5	Keratin, type II cytoskeletal 8	K2C8_HUMAN(KRT8)	53671	5.52	940	22	8.8、11.0	1.75	cytoplasm/keratin filament/nuclear matrix/nucleoplasm	binding; structural	ND
6	Cob(I)yrinic acid a,c-diamide adenosyltransferase, mitochondrial precursor	MMAB_HUMAN (MMAB)	27713	8.86	74	2	>10、>10	0.12	mitochondrion	Binding;enzyme	1; 28
7	Keratin, type II cytoskeletal 8	K2C8_HUMAN (KRT8)	53671	5.52	584	14	>10、>10	0.81	cytoplasm/keratin filament/nuclear matrix/nucleoplasm	binding; structural	ND
8	D-3-phosphoglycerate dehydrogenase	SERA_HUMAN (PHGDH)	57356	6.29	342	9	>10、>10	0.4	cytosol; extracellular vesicular exosome	electron carrier activity; enzyme; binding	1; 4; 5; 22;
9	Gamma-glutamyltranspeptidase 1 precursor	GGT1_HUMAN (GGT1)	61714	6.65	75	1	>10、>10	0.05	cell membrane; single-pass type II membrane protein	Enzyme	1; 17; 25; 21; 14
10	NADH dehydrogenase [ubiquinone] iron-sulfur protein 8, mitochondrial precursor	NDUS8_HUMAN (NDUFS8 )	24203	6	168	4	>10、>10	0.47	mitochondrion	binding; enzyme	1; 2; 6; 11; 19; 29
11	Keratin, type I cytoskeletal 19	K1C19_HUMAN (KRT19)	44065	5.04	932	14	>10、>10	1.75	cytoplasma	structural	ND
12	Ciliary dynein heavy chain 5	DYH5_HUMAN (DNAH5)	532504	5.79	47	11	>10、>10	ND	cytoplasm	enzyme; binding; microtubule motor activity	2
13	Nesprin-2	SYNE2_HUMAN (SYNE2)	801817	5.26	48	3	>10、>10	ND	mitochondrion; nucleus; cytoplasm	Binding	ND
14	Keratin, type I cytoskeletal 10	K1C10_HUMAN (KRT10)	59711	5.13	496	10	>10、>10	0.46	cytoplasm; intermediate filament; mucleus	structural	3;
15	Integrin alpha-L precursor	ITAL_HUMAN (ITGAL)	129942	5.4	45	12	>10、>10	ND	membrane; single-pass type I membrane protein	binding; ICAM-3 receptor activity;	3; 7; 8; 9; 16; 18; 20; 23; 24; 26; 27.

Data from http://www.uniprot.org/uniprot. ND, no data shown in the page of Mascot Search Results

*, data from KEGG database (http://www.kegg.jp/kegg/pathway.html) loading gene name.

1: Metabolic pathways; 2: Huntington's disease;3: *Staphylococcus aureus* infection;4: Biosysthesis of amino acids; 5: Glycine, serine and threonine metabolism; 6: Oxidative phosphorylation; 7: Regulation of actin cytoskeleton; 8: Natural killer cell mediated cytotoxicity; 9: Rap1 signaling pathway; 10: Pathogenic Escherichia coli infection; 11: Non-alcoholic fatty liver disease; 12: Phagosome; 13: Gap junction; 14: Glutathione metabolism; 15: Glycerophospholipid metabolism; 16: Cell adhesion molecules (CAMs); 17: Arachidonic acid metabolism; 18: Viral myocarditis; 19: Alzheimer's disease; 20: Epstein-Barr virus infection; 21: Taurine and hypotaurine metabolism; 22: Carbon metabolism; 23: Malaria; 24: Leukocyte transendothelial migration; 25: Cyanoamino acid metabolism; 26: Rheumatoid arthritis; 27: HTLV-I infection; 28: Porphyrin and chlorophyll metabolism; 29: Parkinson's disease

### Transcriptional profiles of mitochondria proteins were changed by EFV

**Fig 3:**
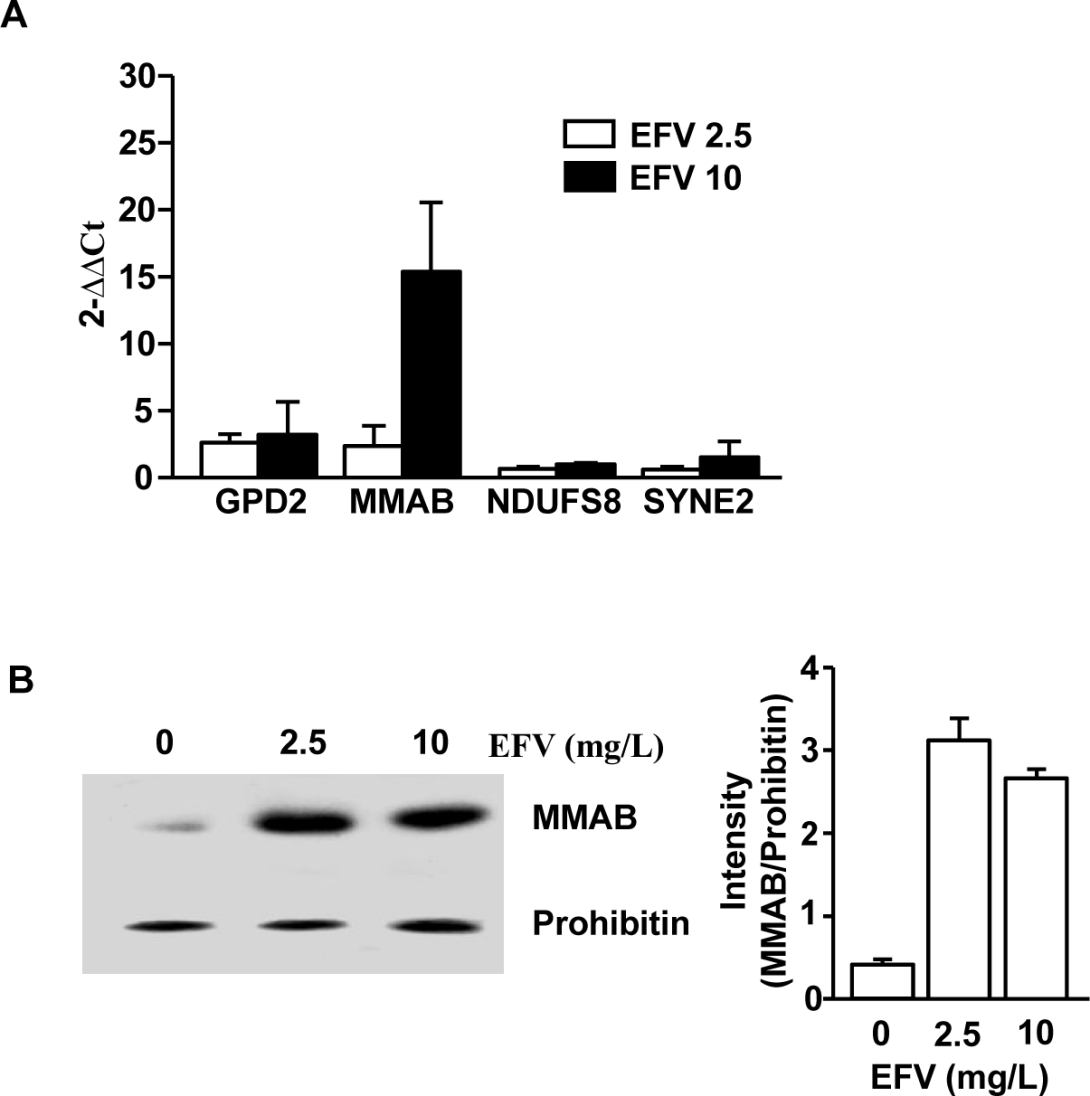
MMAB levels were up-regulated in mitochondria by treatment with EFV. Huh-7 cells were treated with 2.5 or 10 mg/L EFV for 2 h. A) Expression levels of GPD2, MMAB, NDUFS8 and SYNE2 mRNA. B) Expression levels of MMAB in mitochondria. Data are representative of, or the average of analyses of three independent experiments.

Our data that EFV promoted regulation of protein expression in mitochondria prompted us to further examine whether EFV also altered mRNA levels in mitochondria of Huh-7 cells. We analyzed the change of expression levels of 4 mitochondrial genes, including GPD2, MMAB, NDUFS8 and SYNE2, by quantitative real-time PCR in EFV-treated Huh-7 cells. As shown in Table 2 and Fig 3A, EFV promoted substantial dysregulation of those 4 genes compared to the control. In line with protein expression levels, 3 genes in mRNA levels were increased, whereas Glycerol-3-phosphate dehydrogenase (GPD2) was not consistently changed.

Since alteration of MMAB levels was greater than for the other 3 mitochondrial proteins, we next confirmed change of MMAB protein levels in EFV-treated Huh-7 cells by WB analysis. The protein expression levels of MMAB were significantly increased by treatment with EFV 2.5 and EFV 10 (Fig 3B). These data confirmed that EFV treatment of Huh-7 cells promoted up-regulation of MMAB levels in the mitochondria.

### MMAB protein levels were up-regulated in liver of EFV-treated HIV patients

**Fig 4:**
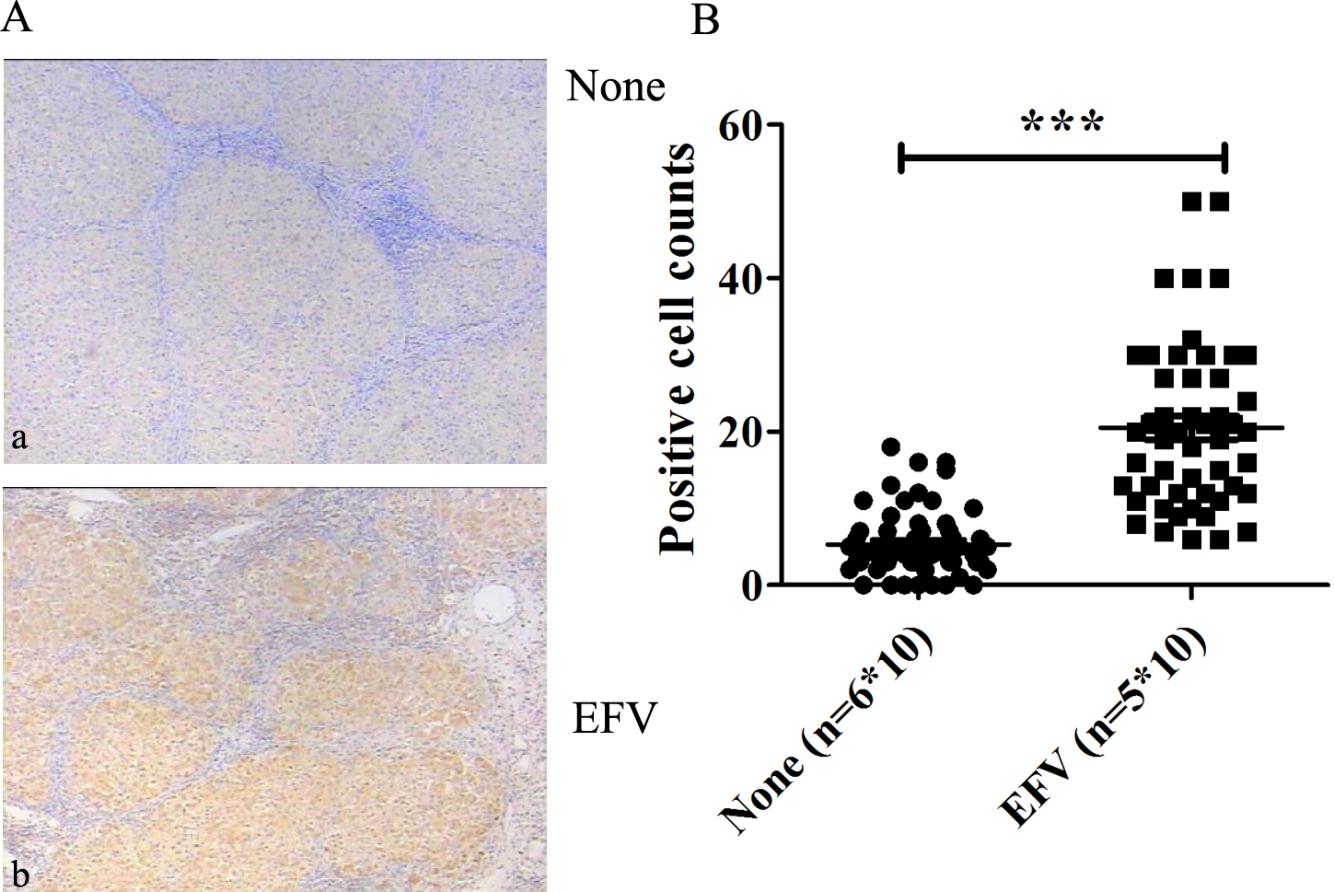
Over-expression of MMAB in the liver of EFV-treated HIV-infected patients. The liver sections from EFV-treated (EFV), or non-treated or PI-treated HIV patients (None) were stained with anti-MMAB monoclonal antibodies. A) Immunohistochemistry analysis. a, from patient without EFV treatment, b, from EFV treated patients. B) Quantitative analysis of MMAB expression in EFV (n = 5*10) or none (n = 6*10). Data are representative of, or the average of analyses of five samples from EFV-treated patients and 6 from patients without treatment or treated with PI drugs. Each sample was randomly scanned for 10 images, and used for statistic analysis.

As a proof of principle, to confirm the relevance of the proteins identified by proteomic analysis in EFV-treated Huh-7 cells, we measured the expression levels of MMAB in human liver tissue from HIV infected patients treated with or without EFV. EFV-treated HIV patients showed marked increases in the MMAB levels in liver compared to HIV patients who were not treated with EFV (Fig 4). Moreover, the quantitative analysis of MMAB expression showed significant up-regulation in the liver from EFV-treated HIV patients compared to the liver of patients without EFV treatment. These data showed that EFV treatment promoted up-regulation of MMAB in the liver of HIV patients.

### Knock-down of MMAB decreased the production of ROS

**Fig 5:**
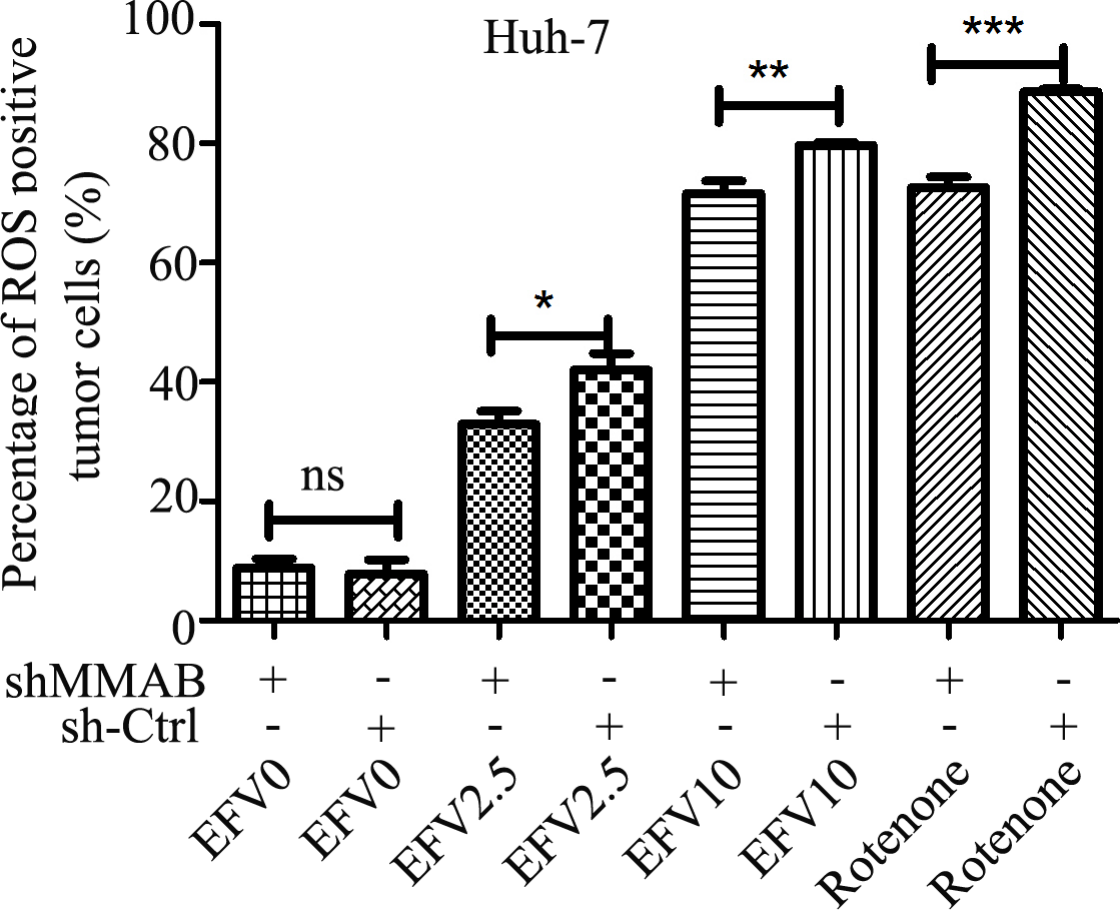
ROS production in Huh-7 cells after EFV treatment with or without MMAB knockdown. The Huh-7 cells were transfected with MMAB-shRNA for 48 h and treated with EFV for additional 2 h. The ROS levels in the cells were measured by flow cytometry. Rotenone was used as positive control. *, **, *** or ****, and n.s. represent *p* < 0.05, 0.01, 0.001 and no significant difference, respectively.

To study the relationship between MMAB and ROS, we measured the production of ROS in MMAB shRNA transfected Huh-7 cells with EFV (0, 2.5, 10 mg/L) or rotenone treatment. As shown in Fig 5, in MMAB shRNA-transfected Huh-7 cells, the level of ROS production was significantly decreased under EFV or rotenone treatment.

### Up-regulation of MMAB by EFV was not a consequence of induced oxidative stress

**Fig 6:**
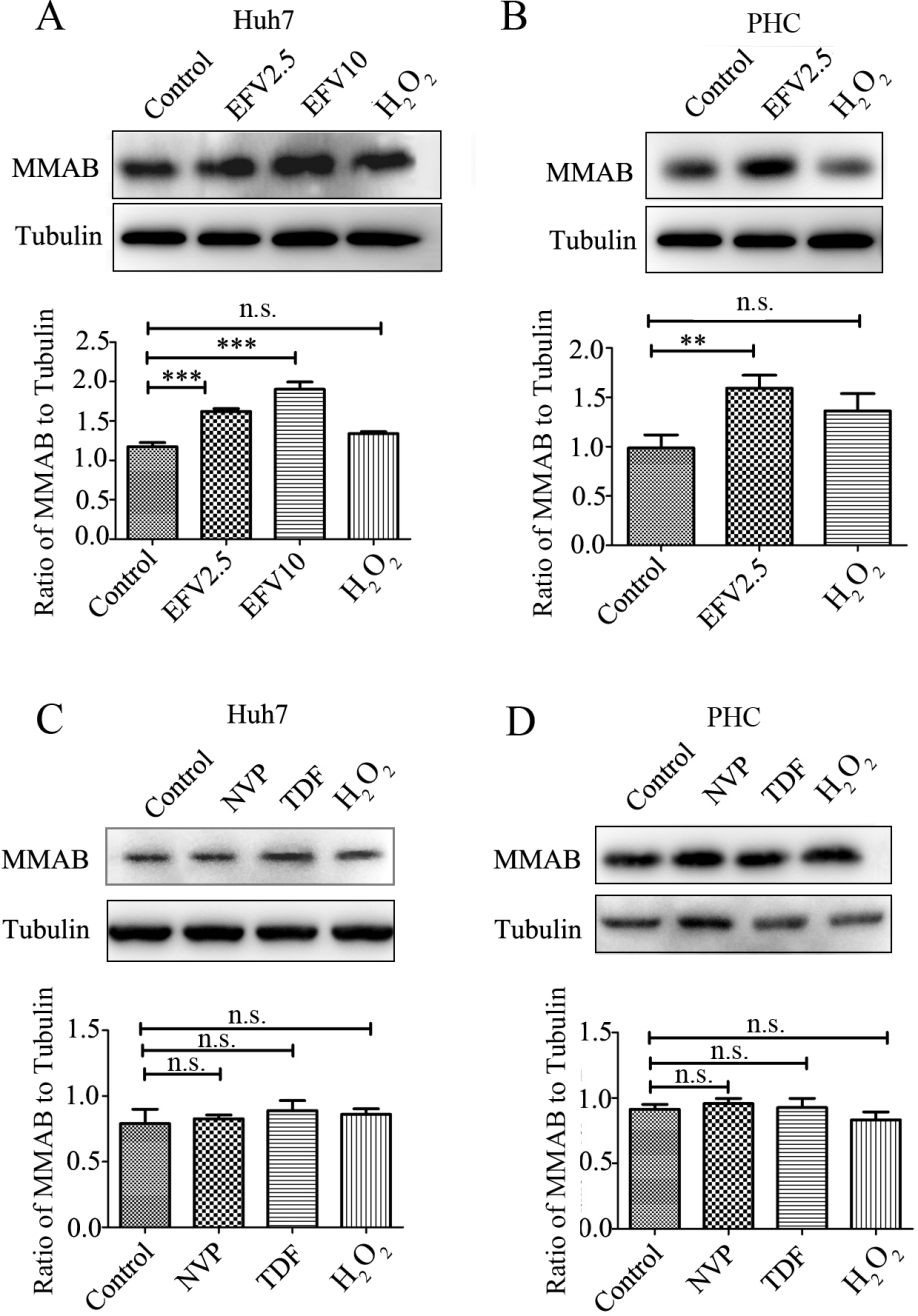
Upregulation of MMAB protein expression levels in Huh7 cells and primary hepatocyte cells by EFV treatment. A) and C) Huh-7 cells; B) and D) primary hepatocyte cells (PHC). A) and B) were treated with 0.1% methanol (Control), EFV and H_2_O_2_ in Huh-7 and PHC for 2 h. C) and D) were treated with NVP, TDF, H_2_O_2_ for 2 h. EFV2.5 and EFV10 represent EFV treated at the concentration of 2.5 mg/L and 10 mg/L. *, **, *** or ****, and ns represent *p* < 0.05, 0.01, 0.001 and no significant difference, respectively.

To detect whether the up-regulation of MMAB was an EFV-specific effect or general effect due to increased oxidant stress we analyzed MMAB expression in EFV or H_2_O_2_ treated Huh-7 cells (Fig 6A) and primary hepatocytes (Fig 6B). MMAB protein expression was significantly higher in EFV treated Huh-7 cells or primary hepatocytes. However, no statistically significant difference was found after H_2_O_2_ treatment, although similar up-regulation of ROS level was detected in EFV or H_2_O_2_ treated Huh-7 cells (Fig 1D) or primary hepatocytes (Fig 1E).

We also looked for an effect of other anti-HIV drugs on MMAB expression. Neither NVP (with hepatotoxicity) nor TDF (without hepatotoxicity) caused a significant change of MMAB expression in Huh-7 cells (Fig 6C) or primary hepatocytes (Fig 6D).

### Knock-down of MMAB promoted up-regulation of β-Alanine

**Fig 7:**
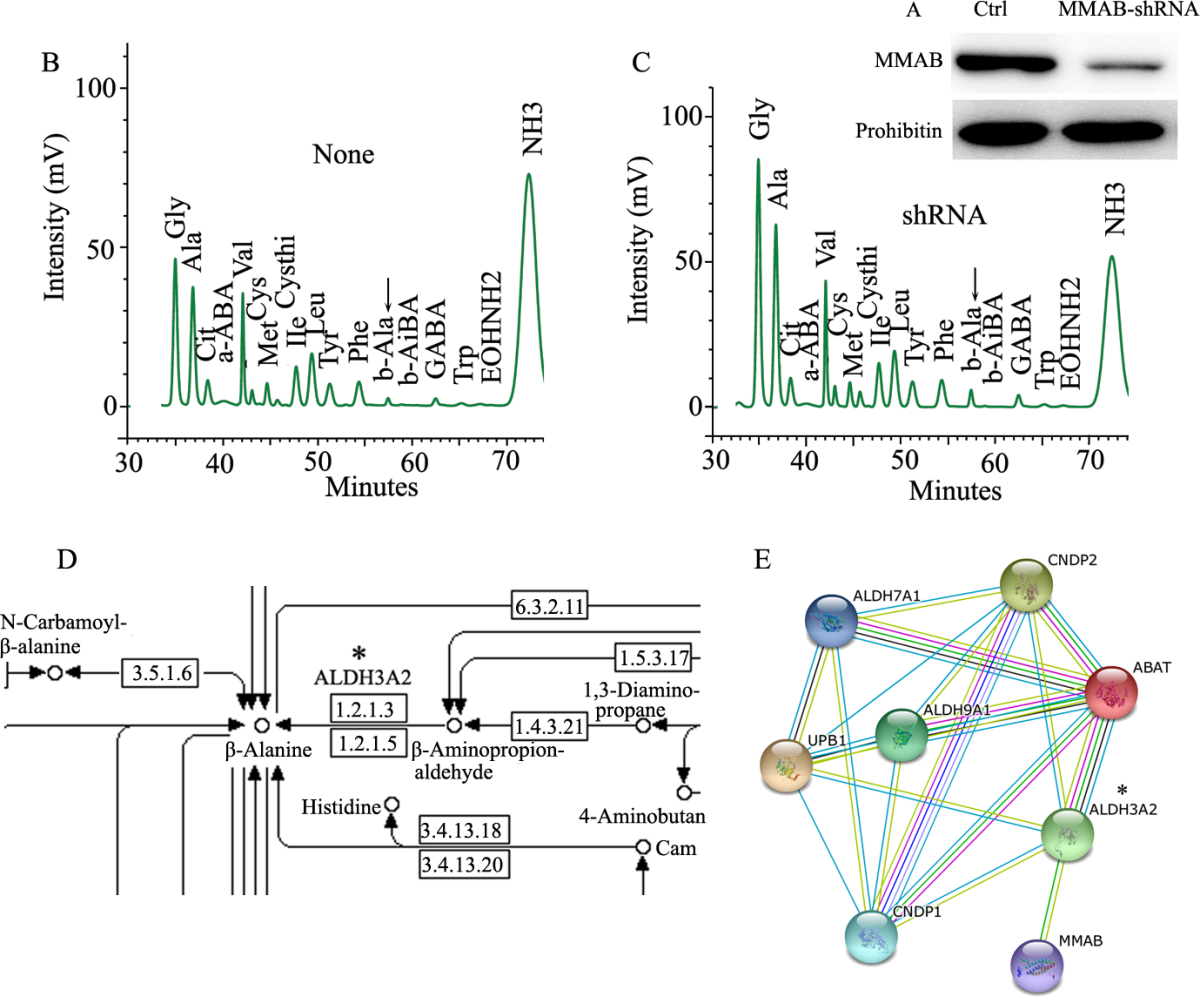
Enhancement of β-Ala production in Huh-7 cells with Knock-down of MMAB. A) Huh-7 cells were transfected with MMAB-shRNA, and cultured for 48 h. B) and C) MMAB-siRNA transfected Huh-7 cells were analyzed for amino acid content and part of the amino acid profile is shown. The differently expressed amino acid (β-Alanine (b-Ala)) is highlighted by the arrow. B) is from control and C) is from shMMAB. D) and E) beta-Alanine metabolism pathway (from map00410 in KEGG) and protein interacting network with MMAB. The direct interacting protein ALDH3A2 is highlighted by “*” (D) and E)).

According to KEGG pathway analysis, MMAB is involved in the pathway of porphyrin and chlorophyll metabolism (Table 3). To further determine the function of MMAB, we examined the metabolism of amino acids in MMAB shRNA transfected Huh-7 cells (Fig 7A). By amino acid analyzer, the MMAB shRNA transfected Huh-7 cells showed substantial increase in β-Alanine (β-Ala) concentration (b-Ala, highlighted by arrow; Fig 5C) compared to un-transfected Huh-7 cells (Fig 7B). Moreover, inspection of the amino acid metabolism pathway indicated that β-Ala metabolism is linked with ALDH3A2 expression (highlighted by star; Fig 5D), a protein interacting with MMAB (Fig 5E).

### Knock-down of MMAB decreased the expression of ALDH3A2

**Fig 8:**
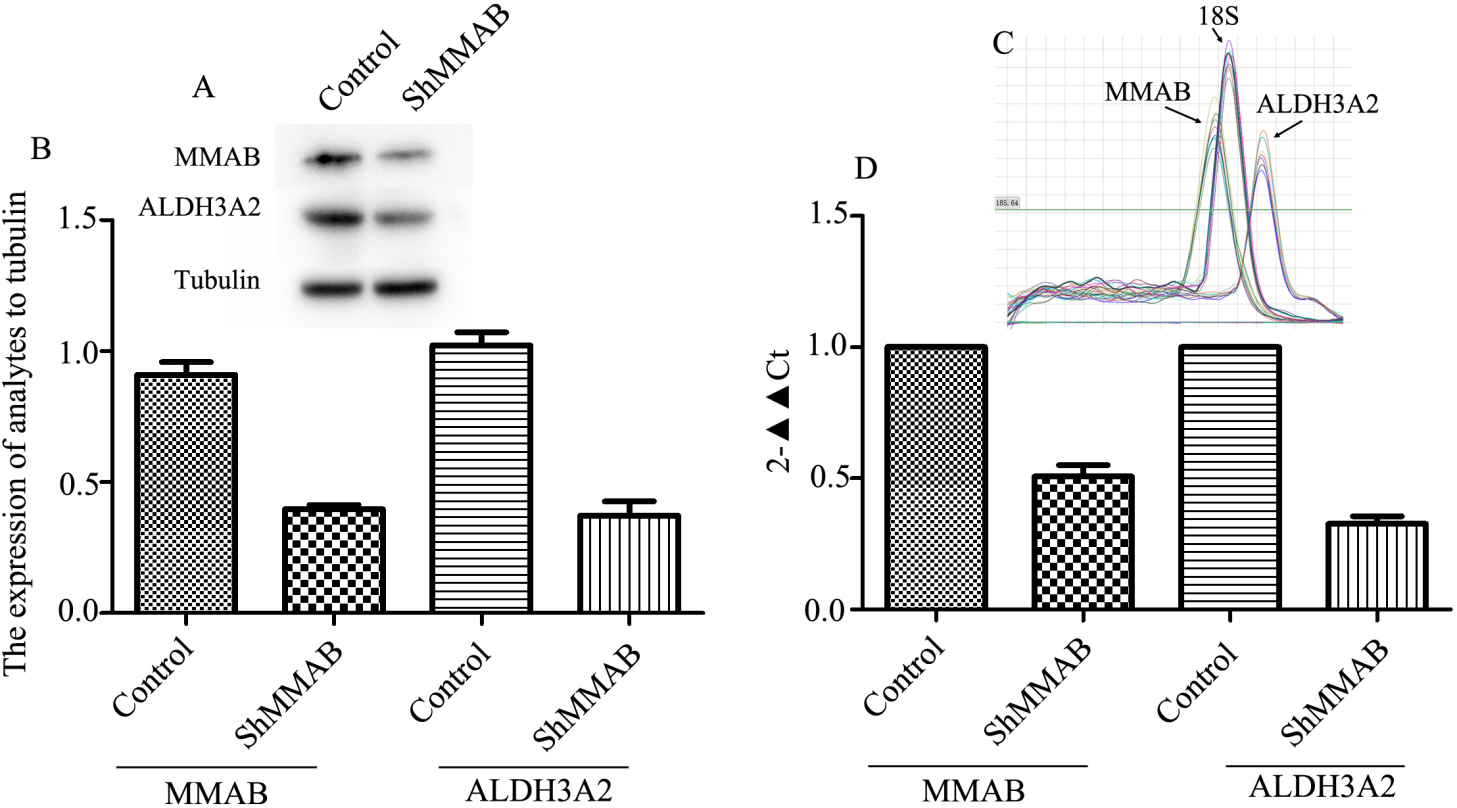
Decreases of ALDH3A2 expression in Huh-7 cells with MMAB Knock-down. Huh-7 cells were transfected with MMAB-shRNA, and cultured for 48 h. A) and B) The protein expressions of MMAB and ALDH3A2 detected by WB. B) statistical analysis in three replicate experiments. Optical density was analyzed by ImageJ software. Each band was read twice. Tubulin was used as reference protein. C) and D) The mRNA expressions of MMAB and ALDH3A2 detected by real-time RT-PCR. C) the fusion curve of MMAB, ALDH3A2 and 18S. D) statistical analysis in three replications. In each experiment, two parallel samples were loaded. 18S was used as reference gene.

Since we suggest MMAB regulate β-Ala production through ALDH3A2, we detected ALDH3A2 expression in MMAB shRNA transfected Huh-7 cells, and found that ALDH3A2 was significantly down-regulated for 64% and 68% in protein level detected by WB and in mRNA level detected by RT-PCR in the MMAB shRNA cells compared with the controls (Fig 8).

## Discussion

**Fig 9:**
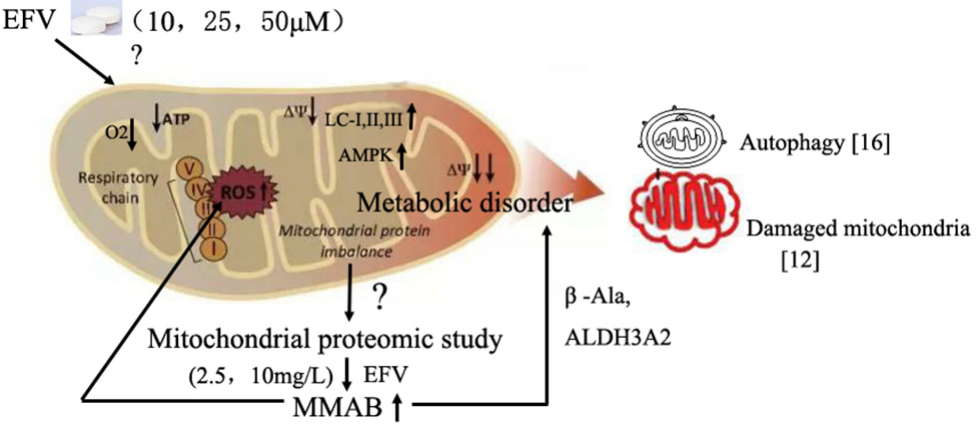
Proposed model for EFV-induced mitotoxicity based on previous studies and our results.

Drug-induced liver injury (DILI) is the primary adverse event that results in the withdrawal of drugs from the market and a frequent reason for the failure of drug candidates in development [29]. EFV-based first-line antiretroviral therapy has a general hepatotoxicity of 3.55% [30]. The mitochondrion is one of the organelles that can become targets for hepatotoxicity involving ROS excessive production, mitochondrial membrane depolarization, *etc*[16] (Fig 9). Recent advances in proteomic technology have provided promising ways to discover and identify novel biomarkers in various fields of clinical medicine [31–33]. The application of various gel-based and gel-free methods has facilitated the discovery of potential clinical biomarkers, although there has been a long and uncertain path from marker discovery to clinical utility. In this study, we followed previous discoveries that EFV-induced hepatotoxicity (Fig 9), and focused to find new biomarkers of EFV-induced hepatotoxicity in Huh-7 cells by proteomic technology, and found that MMAB is a critical mitochondrial protein in the response to EFV treatment in Huh-7. This is the first study of EFV-induced hepatotoxicity through mitochondrial proteomic analysis and we found a potential candidate protein, MMAB, which should be controlled during EFV treatment in patients for preventing hepatotoxicity.

Previous reports showed that EFV induces mitochondrial dysfunction through apoptosis, autophagy or oxidative stress [13, 16] (Fig 9). In this study, after determining the production of ROS by EFV treatment in Huh-7 cells, we analyzed the alteration of protein expression in the mitochondria of Huh-7 cells responding to EFV. We also undertook functional analysis of the differentially expressed proteins through the GO database integrated in SWISS-PROT database. We identified 12 changed proteins to be proteins with binding function, such as tubulin alpha-ubiquitous chain (spot 1), glycerol-3-phosphate dehydrogenase (spot 2), keratin, type II cytoskeletal 8 (spot 3), serum albumin precursor (spot 4) and Cob(I)yrinic acid a,c-diamide adenosyltransferase (spot 6). The others were enzyme or structural proteins (Table 3). Thus, it may be that EFV induces hepatotoxicity mainly through affecting proteins involved in signal delivery in addition to destroying cell structure.

To further understand the function of the differentially expressed proteins identified in this work, we searched the biochemical pathways derived from SWISS-PROT database and KEGG pathway database (http://www.kegg.jp/kegg/pathway.html). Nine of the 13 proteins have been given pathway annotations. Of these, four proteins (MMAB, GGT1, PHGDH and NDUFS8) are involved in a metabolic pathway. MMAB protein levels were remarkably increased by EFV in mitochondria of Huh-7 cells, consistent with increased mRNA expression levels. MMAB is an enzyme which catalyzes the final step in the conversion of vitamin B(12) into adenosylcobalamin (AdoCbl) [34]. Defective synthesis of adenosylcobalamin results in a disorder of methylmalonate and cobalamin metabolism [35, 36]. Moreover, metabolism of vitamin B (12) is required for prevention of cell apoptosis [37, 38]. Since in this study we showed that EFV treatment induces up-regulation of MMAB, it might also affect the metabolism of vitamin B (12). However, further studies are required to establish whether MMAB alters the vitamin B (12) signaling pathway in Huh-7 cells, and to define the relationship between EFV-MMAB and cell apoptosis.

We speculated that EFV may affect cell metabolic pathways through regulating some proteins’ expression and free amino acid levels. Our results showed that β-Ala was up-regulated in the MMAB shRNA transfected Huh-7 cells. Moreover, by STRING analysis, MMAB interacted with ALDH3A2 (aldehyde dehydrogenase 3 family, member A2) which is an upstream signaling molecule of the β-Ala signal pathway. A proposed model for EFV-induced decrease in the mitochondrial membrane function through regulating MMAB-involved metabolic pathways (Fig 9) was built. Although, we found this potentially critical function of MMAB in an amino acid signaling pathway, the role of β-Ala in ROS production and apoptosis of Huh-7 cells must be determined by further study.

In conclusion, the current study demonstrates that increased MMAB level in mitochondria might be a novel biomarker of hepatotoxicity induced by Efavirenz. Given the widespread and life-long use of EFV in AIDS patients, this newly discovered mechanism of cellular insult involving a metabolic pathway could offer some new clues to comprehend the hepatic toxicity. It also points to a potential use of MMAB as a biomarker of hepatotoxicity. Moreover, the modulated proteins identified in this study may provide the basis for the development of specific targeting drugs for treatment of EFV-induced hepatotoxicity.

## Supporting information

S1 Table The peptides of 15 differential protein spots. (DOCX)

S1 Fig Mitochondrial ROS detection induced by EFV. Huh-7 cells were treated with indicated reagent for 2 hours. Mitochondrial ROS was stained by MitoSOX probe and shown in red. Nucleus was stained by Hoechst 33342 and shown in blue. The data is representative from 3 independent results.
(JPG)

S2 Fig The MS/MS of the peptide from Gamma-glutamyltranspeptidase 1 precursor (spot 9). (TIF)
