# Supplementary material for: Increased MMAB level in mitochondria as a novel biomarker of hepatotoxicity induced by Efavirenz
Source: PLoS One. 2017 Nov 30;12(11):e0188366. doi: 10.1371/journal.pone.0188366 (PMC5708658; doi:10.1371/journal.pone.0188366)
Supplement: S1 Table — (DOCX) [file pone.0188366.s001.docx]

S1Table The peptides of 15 differential protein spots

| 1 | TBAK_HUMAN    Mass: 50804    Score: 770    Queries matched: 16   emPAI: 1.26 | | | | | | | | | |
| --- | --- | --- | --- | --- | --- | --- | --- | --- | --- | --- |
|  | Tubulin alpha | | | | | | | | | |
|  | Query | Observed | Mr(expt) | Mr(calc) | Delta | Miss | Score | Expect | Rank | Peptide |
|  | 155 | 444.3800 | 886.7454 | 886.4259 | 0.3196 | 0 | 35 | 0.11 | 1 | K.FDLMYAK.R |
|  | 470 | 508.2900 | 1014.5654 | 1014.5709 | -0.0055 | 0 | 81 | 1.1e-006 | 1 | K.DVNAAIATIK.T |
|  | 475 | 543.3300 | 1084.6454 | 1084.6128 | 0.0326 | 0 | 49 | 0.0015 | 1 | K.EIIDLVLDR.I |
|  | 192 | 470.9800 | 1409.9182 | 1409.7667 | 0.1515 | 0 | 23 | 0.95 | 1 | R.QLFHPEQLITGK.E |
|  | 479 | 744.5000 | 1486.9854 | 1486.8719 | 0.1136 | 0 | 91 | 5.3e-008 | 1 | R.LISQIVSSITASLR.F |
|  | 388 | 792.9500 | 1583.8854 | 1583.7443 | 0.1412 | 0 | 62 | 0.00013 | 1 | R.SIQFVDWCPTGFK.V |
|  | 483 | 851.4800 | 1700.9454 | 1700.8985 | 0.0469 | 0 | 81 | 6.7e-007 | 1 | R.AVFVDLEPTVIDEVR.T |
|  | 254 | 573.8800 | 1718.6182 | 1717.8747 | 0.7435 | 0 | 56 | 0.00084 | 1 | R.NLDIERPTYTNLNR.L |
|  | 447 | 879.5700 | 1757.1254 | 1755.9559 | 1.1695 | 0 | 44 | 0.009 | 1 | R.IHFPLATYAPVISAEK.A |
|  | 484 | 912.9500 | 1823.8854 | 1823.9782 | -0.0927 | 0 | 41 | 0.0059 | 1 | K.VGINYQPPTVVPGGDLAK.V |
|  | 461 | 933.5000 | 1864.9854 | 1863.8971 | 1.0883 | 0 | 95 | 8e-008 | 1 | R.AVCMLSNTTAIAEAWAR.L |
|  | 469 | 1004.5700 | 2007.1254 | 2006.8858 | 0.2396 | 0 | 110 | 2.6e-009 | 1 | K.TIGGGDDSFNTFFSETGAGK.H |
| 2 | GPDM_HUMAN    Mass: 81296    Score: 603    Queries matched: 22   emPAI: 0.37 | | | | | | | | | |
|  | Glycerol-3-phosphate dehydrogenase, mitochondrial precursor | | | | | | | | | |
|  | Query | Observed | Mr(expt) | Mr(calc) | Delta | Miss | Score | Expect | Rank | Peptide |
|  | 360 | 688.8700 | 687.8627 | 687.4353 | 0.4274 | 0 | 20 | 2.8 | 1 | R.LAILMK.T |
|  | 113 | 398.6300 | 795.2454 | 794.4650 | 0.7804 | 0 | 14 | 9 | 1 | R.VPIPVDR.S |
|  | 417 | 409.2300 | 816.4454 | 816.4527 | -0.0073 | 0 | 41 | 0.013 | 1 | R.IVELMGR.E |
|  | 144 | 413.8600 | 825.7054 | 825.4861 | 0.2193 | 0 | 15 | 6.9 | 1 | R.WPIVGVR.L |
|  | 212 | 469.1300 | 936.2454 | 935.4712 | 0.7742 | 0 | 41 | 0.019 | 1 | K.LDIEQYR.M |
|  | 215 | 476.3100 | 950.6054 | 950.5661 | 0.0393 | 1 | 12 | 25 | 1 | R.RVPIPVDR.S |
|  | 235 | 510.7700 | 1019.5254 | 1018.5811 | 0.9443 | 0 | 14 | 9.8 | 2 | R.TVGLFLQGGK.D |
|  | 266 | 573.2700 | 1144.5254 | 1143.6434 | 0.8821 | 0 | 92 | 2.9e-007 | 1 | R.MNLAIALTAAR.Y |
|  | 306 | 616.2900 | 1230.5654 | 1230.5663 | -0.0008 | 0 | 20 | 3.9 | 1 | K.AADCISEPVNR.E |
|  | 449 | 627.8500 | 1253.6854 | 1253.5710 | 0.1144 | 0 | 43 | 0.0059 | 1 | R.NYLSCDVEVR.R |
|  | 315 | 633.2500 | 1264.4854 | 1264.5969 | -0.1114 | 0 | 78 | 5.5e-006 | 1 | R.SMAEDTINAAVK.T + Oxidation (M) |
|  | 345 | 673.2300 | 1344.4454 | 1343.6932 | 0.7522 | 0 | 17 | 6.8 | 4 | R.SEISLLPSDIDR.Y |
|  | 350 | 677.4100 | 1352.8054 | 1351.6806 | 1.1249 | 0 | 30 | 0.31 | 1 | K.LYDLVAGSNCLK.S |
|  | 377 | 716.3500 | 1430.6854 | 1430.6170 | 0.0685 | 0 | 21 | 2.9 | 1 | K.EYACTAVDMISR.R + Oxidation (M) |
|  | 400 | 768.8700 | 1535.7254 | 1535.7403 | -0.0148 | 0 | 38 | 0.033 | 1 | K.CVINATGPFTDSVR.K |
|  | 241 | 523.3100 | 1566.9082 | 1565.7508 | 1.1574 | 1 | 25 | 1.1 | 1 | R.CKDVLTGQEFDVR.A |
|  | 451 | 576.6100 | 1726.8082 | 1726.8122 | -0.0040 | 1 | 26 | 0.22 | 1 | K.TALVERDDFSSGTSSR.S |
|  | 289 | 598.0100 | 1791.0082 | 1789.8747 | 1.1334 | 0 | 21 | 1.6 | 1 | K.LVGAIVYYDGQHNDAR.M |
|  | 453 | 599.9700 | 1796.8882 | 1796.8475 | 0.0407 | 1 | 39 | 0.0099 | 1 | K.AADCISEPVNREPPSR.E |
| 3 | K2C8_HUMAN    Mass: 53671    Score: 941    Queries matched: 18   emPAI: 1.59 | | | | | | | | | |
|  | Keratin, type II cytoskeletal 8 | | | | | | | | | |
|  | Query | Observed | Mr(expt) | Mr(calc) | Delta | Miss | Score | Expect | Rank | Peptide |
|  | 399 | 827.6800 | 826.6727 | 826.4225 | 0.2502 | 0 | 15 | 6.6 | 1 | K.FASFIDK.V |
|  | 444 | 500.8200 | 999.6254 | 999.5600 | 0.0654 | 0 | 60 | 0.00012 | 1 | R.LQAEIEGLK.G |
|  | 450 | 540.2600 | 1078.5054 | 1078.5043 | 0.0011 | 0 | 52 | 0.00077 | 1 | K.AQYEDIANR.S |
|  | 208 | 542.3500 | 1082.6854 | 1081.5920 | 1.0934 | 1 | 42 | 0.018 | 1 | K.FASFIDKVR.F |
|  | 453 | 565.3500 | 1128.6854 | 1128.6138 | 0.0716 | 0 | 68 | 2.1e-005 | 1 | K.LSELEAALQR.A |
|  | 222 | 569.2600 | 1136.5054 | 1136.5713 | -0.0659 | 0 | 53 | 0.0015 | 1 | K.YEELQSLAGK.H |
|  | 228 | 577.3300 | 1152.6454 | 1152.5485 | 0.0970 | 0 | 40 | 0.036 | 1 | R.EYQELMNVK.L |
|  | 232 | 585.8100 | 1169.6054 | 1168.5434 | 1.0621 | 0 | 40 | 0.027 | 1 | R.AEAESMYQIK.Y |
|  | 454 | 587.3400 | 1172.6654 | 1172.6289 | 0.0366 | 0 | 73 | 6.8e-006 | 1 | K.LVSESSDVLPK.- |
|  | 460 | 660.8900 | 1319.7654 | 1319.6642 | 0.1012 | 0 | 96 | 2.7e-008 | 1 | R.SLDMDSIIAEVK.A |
|  | 462 | 671.4000 | 1340.7854 | 1340.7412 | 0.0443 | 1 | 67 | 2.2e-005 | 1 | R.LQAEIEGLKGQR.A |
|  | 310 | 672.8800 | 1343.7454 | 1343.6681 | 0.0774 | 0 | 69 | 3.2e-005 | 1 | R.ASLEAAIADAEQR.G |
|  | 313 | 676.9700 | 1351.9254 | 1351.6693 | 0.2561 | 0 | 56 | 0.00062 | 1 | R.TEMENEFVLIK.K |
|  | 340 | 710.3800 | 1418.7454 | 1418.7405 | 0.0049 | 0 | 91 | 2e-007 | 1 | R.LEGLTDEINFLR.Q |
|  | 357 | 738.4400 | 1474.8654 | 1474.6908 | 0.1746 | 0 | 12 | 19 | 5 | R.LESGMQNMSIHTK.T |
|  | 468 | 599.9900 | 1796.9482 | 1796.8250 | 0.1231 | 1 | 44 | 0.0033 | 1 | K.DVDEAYMNKVELESR.L |
|  | 469 | 652.7100 | 1955.1082 | 1955.0323 | 0.0759 | 1 | 75 | 2.3e-006 | 1 | R.ASLEAAIADAEQRGELAIK.D |
| 4 | ALBU_HUMAN    Mass: 71317    Score: 638    Queries matched: 17   emPAI: 0.80 | | | | | | | | | |
|  | Serum albumin precursor | | | | | | | | | |
|  | Query | Observed | Mr(expt) | Mr(calc) | Delta | Miss | Score | Expect | Rank | Peptide |
|  | 472 | 464.2900 | 926.5654 | 926.4861 | 0.0793 | 0 | 40 | 0.01 | 1 | K.YLYEIAR.R |
|  | 171 | 476.7600 | 951.5054 | 950.4345 | 1.0709 | 0 | 48 | 0.0049 | 1 | K.DLGEENFK.A |
|  | 478 | 480.8000 | 959.5854 | 959.5552 | 0.0302 | 0 | 50 | 0.0013 | 1 | K.FQNALLVR.Y |
|  | 186 | 500.8400 | 999.6654 | 999.5964 | 0.0690 | 0 | 28 | 0.46 | 1 | K.QTALVELVK.H |
|  | 191 | 507.6500 | 1013.2854 | 1012.5917 | 0.6938 | 0 | 30 | 0.3 | 1 | K.LVAASQAALGL.- |
|  | 193 | 509.3500 | 1016.6854 | 1016.5291 | 0.1563 | 0 | 20 | 2.8 | 1 | K.SLHTLFGDK.L |
|  | 488 | 527.7300 | 1053.4454 | 1054.5811 | -1.1356 | 1 | 6 | 26 | 5 | K.KYLYEIAR.R |
|  | 236 | 571.0600 | 1140.1054 | 1140.6866 | -0.5812 | 1 | 26 | 0.5 | 1 | K.KLVAASQAALGL.- |
|  | 239 | 575.3500 | 1148.6854 | 1148.6077 | 0.0777 | 0 | 51 | 0.0035 | 1 | K.LVNEVTEFAK.T |
|  | 280 | 613.6900 | 1225.3654 | 1225.5979 | -0.2324 | 1 | 35 | 0.076 | 1 | R.FKDLGEENFK.A |
|  | 340 | 679.8200 | 1357.6254 | 1357.6224 | 0.0030 | 0 | 81 | 2.1e-006 | 1 | K.AVMDDFAAFVEK.C + Oxidation (M) |
|  | 493 | 756.4600 | 1510.9054 | 1510.8355 | 0.0699 | 0 | 79 | 1e-006 | 1 | K.VPQVSTPTLVEVSR.N |
|  | 436 | 820.5100 | 1639.0054 | 1638.9305 | 0.0750 | 1 | 53 | 0.0013 | 1 | K.KVPQVSTPTLVEVSR.N |
|  | 437 | 820.9400 | 1639.8654 | 1638.7752 | 1.0902 | 0 | 40 | 0.023 | 1 | K.DVFLGMFLYEYAR.R + Oxidation (M) |
|  | 497 | 682.3800 | 2044.1182 | 2044.0881 | 0.0301 | 0 | 56 | 0.00016 | 1 | K.VFDEFKPLVEEPQNLIK.Q |
| 5 | K2C8_HUMAN    Mass: 53671    Score: 940    Queries matched: 22   emPAI: 1.75 | | | | | | | | | |
|  | Keratin, type II cytoskeletal 8 | | | | | | | | | |
|  | Query | Observed | Mr(expt) | Mr(calc) | Delta | Miss | Score | Expect | Rank | Peptide |
|  | 414 | 500.7700 | 999.5254 | 999.5600 | -0.0346 | 0 | 42 | 0.0085 | 1 | R.LQAEIEGLK.G |
|  | 206 | 515.8400 | 1029.6654 | 1029.5607 | 0.1047 | 0 | 22 | 2.2 | 1 | K.WSLLQQQK.T |
|  | 417 | 540.2400 | 1078.4654 | 1078.5043 | -0.0389 | 0 | 53 | 0.00061 | 1 | K.AQYEDIANR.S |
|  | 227 | 540.3100 | 1078.6054 | 1078.5294 | 0.0760 | 0 | 34 | 0.11 | 1 | R.QLYEEEIR.E |
|  | 419 | 565.3600 | 1128.7054 | 1128.6138 | 0.0916 | 0 | 65 | 3.6e-005 | 1 | K.LSELEAALQR.A |
|  | 245 | 569.2400 | 1136.4654 | 1136.5713 | -0.1059 | 0 | 71 | 2.3e-005 | 1 | K.YEELQSLAGK.H |
|  | 248 | 577.2600 | 1152.5054 | 1152.5485 | -0.0430 | 0 | 53 | 0.0018 | 1 | R.EYQELMNVK.L |
|  | 253 | 585.2800 | 1168.5454 | 1168.5434 | 0.0021 | 0 | 34 | 0.11 | 1 | R.AEAESMYQIK.Y |
|  | 421 | 587.3400 | 1172.6654 | 1172.6289 | 0.0366 | 0 | 75 | 3.7e-006 | 1 | K.LVSESSDVLPK.- |
|  | 309 | 660.8800 | 1319.7454 | 1319.6642 | 0.0812 | 0 | 94 | 1.3e-007 | 1 | R.SLDMDSIIAEVK.A |
|  | 316 | 671.7300 | 1341.4454 | 1340.7412 | 0.7043 | 1 | 25 | 0.83 | 1 | R.LQAEIEGLKGQR.A |
|  | 317 | 672.8200 | 1343.6254 | 1343.6681 | -0.0426 | 0 | 79 | 3.8e-006 | 1 | R.ASLEAAIADAEQR.G |
|  | 424 | 676.9000 | 1351.7854 | 1351.6693 | 0.1161 | 0 | 74 | 4.5e-006 | 1 | R.TEMENEFVLIK.K |
|  | 345 | 710.4200 | 1418.8254 | 1418.7405 | 0.0849 | 0 | 71 | 2.1e-005 | 1 | R.LEGLTDEINFLR.Q |
|  | 359 | 738.4200 | 1474.8254 | 1474.6908 | 0.1346 | 0 | 12 | 18 | 2 | R.LESGMQNMSIHTK.T |
|  | 196 | 494.6100 | 1480.8082 | 1479.7643 | 1.0439 | 1 | 8 | 41 | 8 | R.TEMENEFVLIKK.D |
|  | 268 | 600.0200 | 1797.0382 | 1796.8250 | 0.2131 | 1 | 55 | 0.00081 | 1 | K.DVDEAYMNKVELESR.L |
|  | 428 | 652.7700 | 1955.2882 | 1955.0323 | 0.2559 | 1 | 58 | 7.2e-005 | 1 | R.ASLEAAIADAEQRGELAIK.D |
|  | 416 | 1054.9600 | 2107.9054 | 2108.0056 | -0.1001 | 0 | 26 | 0.45 | 1 | R.ELQSQISDTSVVLSMDNSR.S |
| 6 | MMAB_HUMAN    Mass: 27713    Score: 74     Queries matched: 2   emPAI: 0.12 | | | | | | | | | |
|  | Cob(I)yrinicacida,c-diamideadenosyltransferase, mitochondrial precursor | | | | | | | | | |
|  | Query | Observed | Mr(expt) | Mr(calc) | Delta | Miss | Score | Expect | Rank | Peptide |
|  | 231 | 580.3000 | 1158.5854 | 1158.5669 | 0.0185 | 0 | 16 | 9.4 | 1 | K.GHTFAEELQK.I |
|  | 245 | 599.8900 | 1197.7654 | 1197.6394 | 0.1261 | 0 | 59 | 0.00033 | 1 | R.LSDYLFTLAR.Y |
| 7 | K2C8_HUMAN    Mass: 53671    Score: 584    Queries matched: 14   emPAI: 0.81 | | | | | | | | | |
|  | Keratin, type II cytoskeletal 8 | | | | | | | | | |
|  | Query | Observed | Mr(expt) | Mr(calc) | Delta | Miss | Score | Expect | Rank | Peptide |
|  | 196 | 500.8200 | 999.6254 | 999.5600 | 0.0654 | 0 | 42 | 0.018 | 1 | R.LQAEIEGLK.G |
|  | 218 | 540.2600 | 1078.5054 | 1078.5294 | -0.0240 | 0 | 36 | 0.08 | 1 | R.QLYEEEIR.E |
|  | 219 | 540.2700 | 1078.5254 | 1078.5043 | 0.0211 | 0 | 57 | 0.00063 | 1 | K.AQYEDIANR.S |
|  | 229 | 565.3400 | 1128.6654 | 1128.6138 | 0.0516 | 0 | 60 | 0.00036 | 1 | K.LSELEAALQR.A |
|  | 232 | 569.3700 | 1136.7254 | 1136.5713 | 0.1541 | 0 | 39 | 0.03 | 1 | K.YEELQSLAGK.H |
|  | 236 | 577.3500 | 1152.6854 | 1152.5485 | 0.1370 | 0 | 38 | 0.056 | 1 | R.EYQELMNVK.L |
|  | 243 | 587.2700 | 1172.5254 | 1172.6289 | -0.1034 | 0 | 62 | 0.00026 | 1 | K.LVSESSDVLPK.- |
|  | 292 | 660.8300 | 1319.6454 | 1319.6642 | -0.0188 | 0 | 66 | 6.8e-005 | 1 | R.SLDMDSIIAEVK.A |
|  | 157 | 448.1600 | 1341.4582 | 1340.7412 | 0.7170 | 1 | 14 | 13 | 1 | R.LQAEIEGLKGQR.A |
|  | 299 | 673.1000 | 1344.1854 | 1343.6681 | 0.5174 | 0 | 50 | 0.0025 | 1 | R.ASLEAAIADAEQR.G |
|  | 301 | 676.9800 | 1351.9454 | 1351.6693 | 0.2761 | 0 | 19 | 2.9 | 1 | R.TEMENEFVLIK.K |
|  | 392 | 710.3700 | 1418.7254 | 1418.7405 | -0.0151 | 0 | 67 | 1.9e-005 | 1 | R.LEGLTDEINFLR.Q |
|  | 287 | 652.9600 | 1955.8582 | 1955.0323 | 0.8259 | 1 | 40 | 0.02 | 1 | R.ASLEAAIADAEQRGELAIK.D |
| 8 | SERA_HUMAN    Mass: 57356    Score: 342    Queries matched: 9   emPAI: 0.40 | | | | | | | | | |
|  | D-3-phosphoglycerate dehydrogenase | | | | | | | | | |
|  | Query | Observed | Mr(expt) | Mr(calc) | Delta | Miss | Score | Expect | Rank | Peptide |
|  | 178 | 450.3500 | 898.6854 | 898.5600 | 0.1255 | 0 | 24 | 1.3 | 1 | K.TLGILGLGR.I |
|  | 210 | 494.2500 | 986.4854 | 985.5960 | 0.8894 | 0 | 22 | 2.4 | 1 | R.DLPLLLFR.T |
|  | 243 | 536.2700 | 1070.5254 | 1070.5720 | -0.0466 | 0 | 32 | 0.24 | 1 | K.QADVNLVNAK.L |
|  | 251 | 550.3300 | 1098.6454 | 1098.6033 | 0.0421 | 0 | 64 | 0.00016 | 1 | R.GGIVDEGALLR.A |
|  | 261 | 565.8000 | 1129.5854 | 1129.5979 | -0.0124 | 0 | 45 | 0.008 | 1 | K.VTADVINAAEK.L |
|  | 295 | 649.9300 | 1297.8454 | 1297.7242 | 0.1213 | 0 | 27 | 0.51 | 1 | K.ILQDGGLQVVEK.Q |
|  | 311 | 673.4400 | 1344.8654 | 1344.7613 | 0.1042 | 0 | 40 | 0.036 | 1 | K.GTIQVITQGTSLK.N |
|  | 326 | 717.8600 | 1433.7054 | 1433.6643 | 0.0412 | 0 | 18 | 4.8 | 1 | K.VLISDSLDPCCR.K |
|  | 337 | 744.9100 | 1487.8054 | 1487.7216 | 0.0838 | 0 | 70 | 2.6e-005 | 1 | R.AGTGVDNVDLEAATR.K |
| 9 | GGT1_HUMAN    Mass: 61714    Score: 75     Queries matched: 1   emPAI: 0.05 | | | | | | | | | |
|  | Gamma-glutamyltranspeptidase 1 precursor | | | | | | | | | |
|  | Query | Observed | Mr(expt) | Mr(calc) | Delta | Miss | Score | Expect | Rank | Peptide |
|  | 264 | 701.4200 | 1400.8254 | 1400.7259 | 0.0995 | 0 | 75 | 1.1e-005 | 1 | R.NIDQAVTAALETR.H |
| 10 | NDUS8_HUMAN    Mass: 24203    Score: 168    Queries matched: 4   emPAI: 0.47 | | | | | | | | | |
|  | NADH dehydrogenase [ubiquinone] iron-sulfur protein 8, mitochondrial precursor | | | | | | | | | |
|  | Query | Observed | Mr(expt) | Mr(calc) | Delta | Miss | Score | Expect | Rank | Peptide |
|  | 296 | 589.4300 | 1176.8454 | 1177.6495 | -0.8041 | 0 | 59 | 0.00053 | 1 | R.TLLWTELFR.G |
|  | 322 | 654.8600 | 1307.7054 | 1307.6397 | 0.0657 | 0 | 26 | 0.61 | 1 | R.EPATINYPFEK.G |
|  | 397 | 1021.5600 | 2041.1054 | 2040.9972 | 0.1082 | 0 | 84 | 1e-006 | 1 | K.LCEAICPAQAITIEAEPR.A |
| 11 | K1C19_HUMAN    Mass: 44065    Score: 932    Queries matched: 14   emPAI: 1.75 | | | | | | | | | |
|  | Keratin, type I cytoskeletal 19 | | | | | | | | | |
|  | Query | Observed | Mr(expt) | Mr(calc) | Delta | Miss | Score | Expect | Rank | Peptide |
|  | 435 | 404.2000 | 806.3854 | 806.3923 | -0.0068 | 0 | 39 | 0.016 | 1 | R.LAADDFR.T |
|  | 163 | 425.6900 | 849.3654 | 849.4497 | -0.0843 | 0 | 53 | 0.0015 | 1 | R.FGPGVAFR.A |
|  | 241 | 515.3300 | 1028.6454 | 1028.5866 | 0.0589 | 0 | 67 | 8.1e-005 | 1 | R.VLDELTLAR.T |
|  | 497 | 537.3300 | 1072.6454 | 1072.5876 | 0.0578 | 0 | 73 | 7.2e-006 | 1 | K.ILGATIENSR.I |
|  | 264 | 552.8500 | 1103.6854 | 1103.5393 | 0.1461 | 0 | 41 | 0.021 | 1 | K.LTMQNLNDR.L |
|  | 498 | 561.8200 | 1121.6254 | 1121.5717 | 0.0538 | 0 | 66 | 3.1e-005 | 1 | R.LEQEIATYR.S |
|  | 292 | 602.2700 | 1202.5254 | 1203.5918 | -1.0663 | 0 | 71 | 3.6e-005 | 1 | R.MSVEADINGLR.R |
|  | 302 | 611.8300 | 1221.6454 | 1221.6353 | 0.0101 | 1 | 61 | 0.00018 | 1 | R.TKFETEQALR.M |
|  | 500 | 614.3100 | 1226.6054 | 1226.5891 | 0.0164 | 0 | 41 | 0.0091 | 1 | K.NHEEEISTLR.G |
|  | 502 | 622.3600 | 1242.7054 | 1242.6455 | 0.0599 | 0 | 97 | 2.3e-008 | 1 | R.ALEAANGELEVK.I |
|  | 503 | 677.8400 | 1353.6654 | 1353.5983 | 0.0672 | 0 | 68 | 1.6e-005 | 1 | R.SQYEVMAEQNR.K |
|  | 361 | 683.3800 | 1364.7454 | 1364.7048 | 0.0407 | 1 | 70 | 2.6e-005 | 1 | K.SRLEQEIATYR.S |
|  | 504 | 695.3400 | 1388.6654 | 1388.6783 | -0.0129 | 0 | 91 | 7.9e-008 | 1 | K.AALEDTLAETEAR.F |
|  | 505 | 777.8800 | 1553.7454 | 1553.7434 | 0.0020 | 0 | 95 | 2.6e-008 | 1 | R.QSSATSSFGGLGGGSVR.F |
| 12 | DDX5_HUMAN    Mass: 69618    Score: 50     Queries matched: 3 | | | | | | | | | |
|  | Probable ATP-dependent RNA helicase DDX5 | | | | | | | | | |
|  | Query | Observed | Mr(expt) | Mr(calc) | Delta | Miss | Score | Expect | Rank | Peptide |
|  | 6 | 311.6500 | 621.2854 | 620.3064 | 0.9790 | 1 | 26 | 0.72 | 1 | R.GRGGMK.D + Oxidation (M) |
|  | 204 | 477.2000 | 952.3854 | 953.4832 | -1.0977 | 1 | 14 | 18 | 1 | K.RGGFNTFR.D |
|  | 171 | 436.8500 | 1307.5282 | 1306.7133 | 0.8149 | 1 | 10 | 30 | 4 | K.ENKTIVFVETK.R |
| 13 | SYNE2_HUMAN    Score: 48     Queries matched: 3 | | | | | | | | | |
|  | Nesprin-2 (Nuclear envelope spectrin repeat protein 2) | | | | | | | | | |
|  | Query | Observed | Mr(expt) | Mr(calc) | Delta | Miss | Score | Expect | Rank | Peptide |
|  | 14 | 325.4500 | 973.3282 | 973.5444 | -0.2162 | 0 | 17 | 5.2 | 1 | R.LSLQDGTLK.K |
|  | 70 | 401.0000 | 1199.9782 | 1199.6081 | 0.3701 | 1 | 18 | 4.9 | 2 | K.ADPRALLECR.R |
|  | 205 | 936.9800 | 1871.9454 | 1873.0416 | -1.0962 | 1 | 17 | 3.8 | 1 | K.LVSKTQLEMNLPLMIK.K + Oxidation (M) |
| 14 | K1C10_HUMAN    Mass: 59711    Score: 496    Queries matched: 10   emPAI: 0.46 | | | | | | | | | |
|  | Keratin, type I cytoskeletal 10 | | | | | | | | | |
|  | Query | Observed | Mr(expt) | Mr(calc) | Delta | Miss | Score | Expect | Rank | Peptide |
|  | 149 | 516.5100 | 1031.0054 | 1030.5910 | 0.4144 | 0 | 48 | 0.007 | 1 | R.VLDELTLTK.A |
|  | 159 | 583.3900 | 1164.7654 | 1164.5775 | 0.1880 | 0 | 59 | 0.0003 | 1 | R.LENEIQTYR.S |
|  | 185 | 631.9600 | 1261.9054 | 1261.5899 | 0.3156 | 0 | 50 | 0.003 | 1 | R.SLLEGEGSSGGGGR.G |
|  | 282 | 679.4100 | 1356.8054 | 1356.7110 | 0.0945 | 1 | 11 | 9.3 | 4 | R.QSVEADINGLRR.V |
|  | 216 | 683.3300 | 1364.6454 | 1364.6320 | 0.0134 | 0 | 30 | 0.31 | 1 | R.SQYEQLAEQNR.K |
|  | 283 | 691.3900 | 1380.7654 | 1380.6408 | 0.1246 | 0 | 73 | 5.3e-006 | 1 | R.ALEESNYELEGK.I |
|  | 220 | 695.8500 | 1389.6854 | 1389.6736 | 0.0119 | 0 | 30 | 0.23 | 1 | K.QSLEASLAETEGR.Y |
|  | 285 | 747.3900 | 1492.7654 | 1492.7270 | 0.0385 | 1 | 63 | 4.9e-005 | 1 | R.SQYEQLAEQNRK.D |
|  | 286 | 854.3900 | 1706.7654 | 1706.7649 | 0.0005 | 0 | 77 | 1.6e-006 | 1 | K.GSLGGGFSSGGFSGGSFSR.G |
|  | 288 | 998.9900 | 1995.9654 | 1995.9636 | 0.0018 | 0 | 55 | 0.0002 | 1 | K.ELTTEIDNNIEQISSYK.S |
| 15 | ITAL_HUMAN    Mass: 129942   Score: 45     Queries matched: 13 | | | | | | | | | |
|  | Integrin alpha-L precursor | | | | | | | | | |
|  | Query | Observed | Mr(expt) | Mr(calc) | Delta | Miss | Score | Expect | Rank | Peptide |
|  | 52 | 361.0700 | 720.1254 | 721.2701 | -1.1446 | 0 | 15 | 15 | 2 | K.NCGEDK.K |
|  | 76 | 391.3300 | 780.6454 | 781.4044 | -0.7590 | 0 | 30 | 0.47 | 1 | K.ILDFMK.D + Oxidation (M) |
