# Supplementary figures and images for: Increased MMAB level in mitochondria as a novel biomarker of hepatotoxicity induced by Efavirenz
Source: PLoS One. 2017 Nov 30;12(11):e0188366. doi: 10.1371/journal.pone.0188366 (PMC5708658; doi:10.1371/journal.pone.0188366)

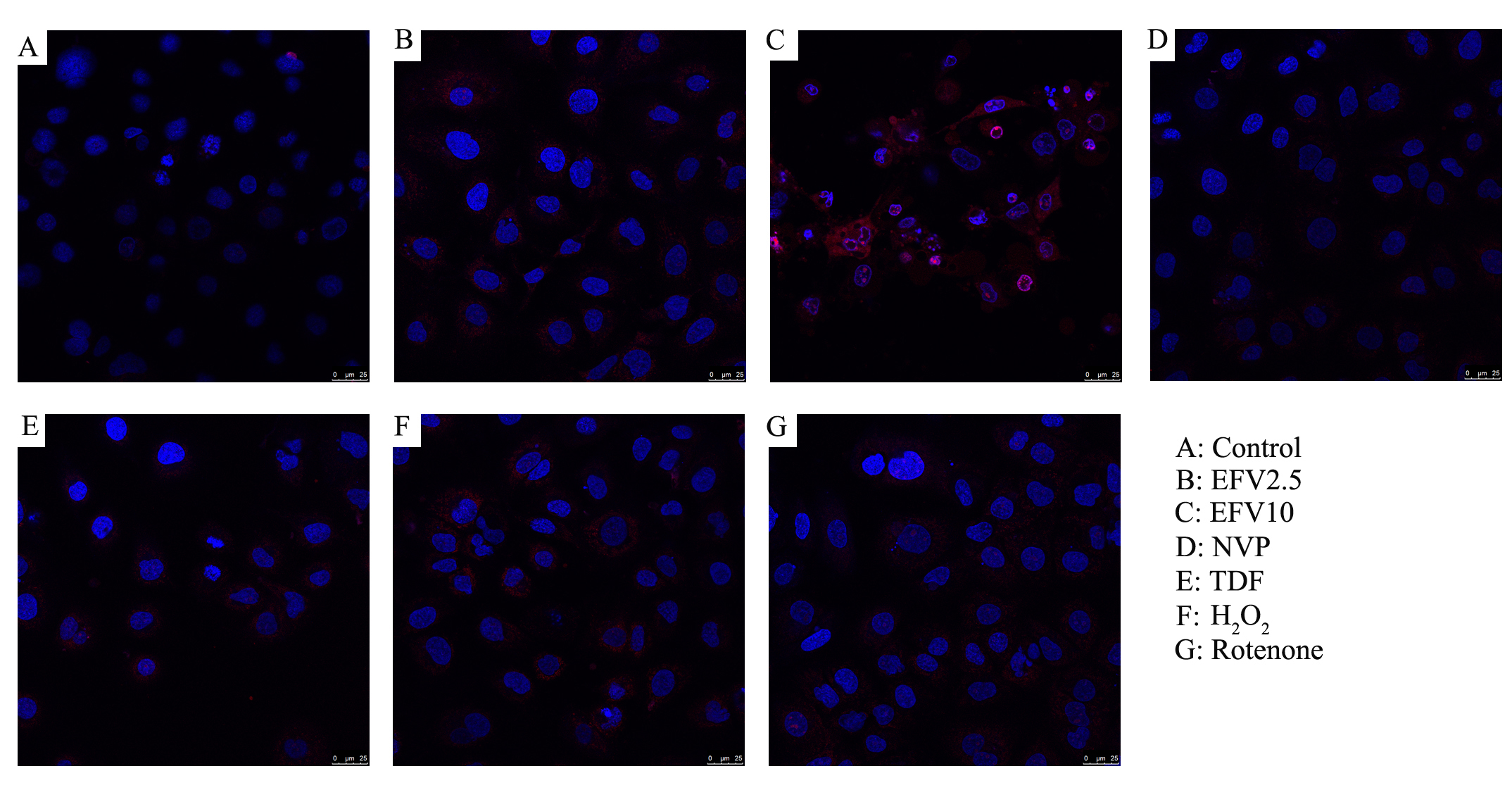

Supplement: S1 Fig — Huh-7 cells were treated with indicated reagent for 2 hours. Mitochondrial ROS was stained by MitoSOX probe and shown in red. Nucleus was stained by Hoechst 33342 and shown in blue. The data is representative from 3 independent results. (JPG) [file pone.0188366.s002.jpg]

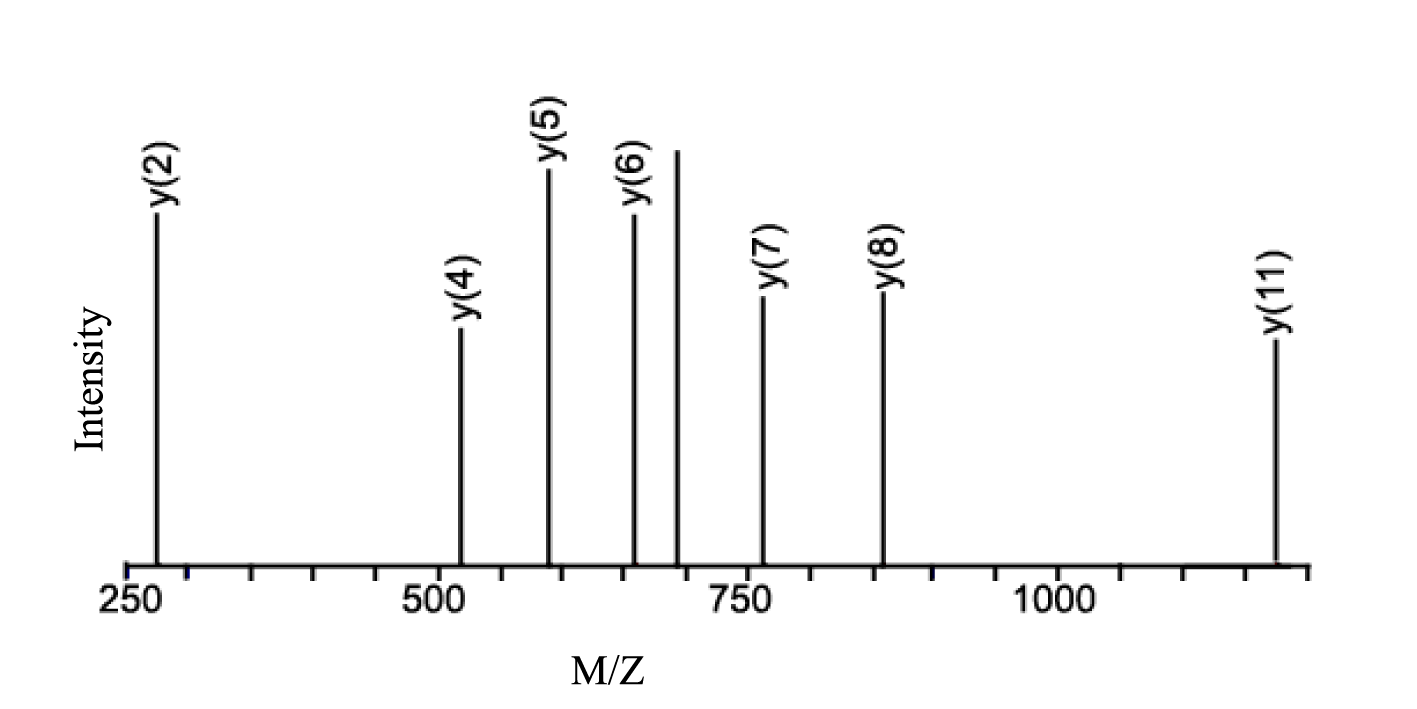

Supplement: S2 Fig — (TIF) [file pone.0188366.s003.tif]
